# CELF Family RNA–Binding Protein UNC-75 Regulates Two Sets of Mutually Exclusive Exons of the *unc-32* Gene in Neuron-Specific Manners in *Caenorhabditis elegans*

**DOI:** 10.1371/journal.pgen.1003337

**Published:** 2013-02-28

**Authors:** Hidehito Kuroyanagi, Yohei Watanabe, Masatoshi Hagiwara

**Affiliations:** 1 Graduate School of Biomedical Science, Tokyo Medical and Dental University, Tokyo, Japan; 2 Medical Research Institute, Tokyo Medical and Dental University, Tokyo, Japan; 3 PRESTO, Japan Science and Technology Agency (JST), Kawaguchi, Japan; 4 Graduate School of Medicine Kyoto University, Kyoto, Japan; University of Colorado Boulder, United States of America

## Abstract

An enormous number of alternative pre–mRNA splicing patterns in multicellular organisms are coordinately defined by a limited number of regulatory proteins and *cis* elements. Mutually exclusive alternative splicing should be strictly regulated and is a challenging model for elucidating regulation mechanisms. Here we provide models of the regulation of two sets of mutually exclusive exons, 4a–4c and 7a–7b, of the *Caenorhabditis elegans uncoordinated (unc)-32* gene, encoding the *a* subunit of V_0_ complex of vacuolar-type H^+^-ATPases. We visualize selection patterns of exon 4 and exon 7 *in vivo* by utilizing a trio and a pair of symmetric fluorescence splicing reporter minigenes, respectively, to demonstrate that they are regulated in tissue-specific manners. Genetic analyses reveal that RBFOX family RNA–binding proteins ASD-1 and FOX-1 and a UGCAUG stretch in intron 7b are involved in the neuron-specific selection of exon 7a. Through further forward genetic screening, we identify UNC-75, a neuron-specific CELF family RNA–binding protein of unknown function, as an essential regulator for the exon 7a selection. Electrophoretic mobility shift assays specify a short fragment in intron 7a as the recognition site for UNC-75 and demonstrate that UNC-75 specifically binds via its three RNA recognition motifs to the element including a UUGUUGUGUUGU stretch. The UUGUUGUGUUGU stretch in the reporter minigenes is actually required for the selection of exon 7a in the nervous system. We compare the amounts of partially spliced RNAs in the wild-type and *unc-75* mutant backgrounds and raise a model for the mutually exclusive selection of *unc-32* exon 7 by the RBFOX family and UNC-75. The neuron-specific selection of *unc-32* exon 4b is also regulated by UNC-75 and the *unc-75* mutation suppresses the Unc phenotype of the exon-4b-specific allele of *unc-32* mutants. Taken together, UNC-75 is the neuron-specific splicing factor and regulates both sets of the mutually exclusive exons of the *unc-32* gene.

## Author Summary

Tissue-specific and mutually exclusive alternative pre–mRNA splicing is a challenging model for elucidating regulation mechanisms. We previously demonstrated that evolutionarily conserved RBFOX family RNA–binding proteins ASD-1 and FOX-1 and a muscle-specific RNA–binding protein SUP-12 cooperatively direct muscle-specific selection of exon 5B of the *C. elegans egl-15* gene. Here we demonstrate that two sets of mutually exclusive exons, 4a–4c and 7a–7b, of the *unc-32* gene are regulated in tissue-specific manners and that ASD-1 and FOX-1, expressed in a variety of tissues, can regulate the neuron-specific selection of *unc-32* exon 7a in combination with the neuron-specific CELF family RNA–binding protein UNC-75. We determine the *cis*-elements for the RBFOX family and UNC-75, which separately reside in intron 7b and intron 7a, respectively. By analyzing the partially spliced RNA species, we propose the orders of intron removal and the sites of action for the RBFOX family and UNC-75 in the mutually exclusive selection of exon 7a and exon 7b. We also demonstrate that UNC-75 regulates the neuron-specific selection of exon 4b and propose the models of the mutually exclusive selection of exons 4a, 4b, and 4c. These studies thus provide novel modes of regulation for tissue-specific and mutually exclusive alternative splicing *in vivo*.

## Introduction

Alternative splicing of pre-mRNAs is a major source of proteomic complexity in metazoans. More than 90% of human multi-exon genes undergo alternative pre-mRNA processing and many alternative splicing events are controlled in tissue- and cell-type dependent manners [1]. Mis-splicing of pre-mRNAs underlie many inherited diseases [2]. A variety of auxiliary *trans*-acting factors and *cis*-acting elements regulating alternative splicing have been identified [3], [4], [5], [6]. Recent genome-wide studies of protein-RNA interactions for *trans*-acting splicing factors led to creation of RNA splicing maps [7]. Combinations of hundreds of RNA features were used to assemble ‘splicing codes’ to predict splicing patterns in four major tissues to a significant extent [8]. However, much of our knowledge of splicing regulation relies on experiments utilizing cultured cells, and therefore complex mechanisms of the tissue-specific regulation of pre-mRNA splicing by coordination of multiple *trans*-factors and *cis*-elements in living organisms remain less understood.

Mutually exclusive splicing should consist of multiple steps of strictly regulated splicing events and offers good models for elucidating regulation mechanisms for alternative pre-mRNA splicing [9], [10]. Among them, fibroblast growth factor receptor (FGFR) genes have been well studied because tissue-specific and mutually exclusive selection of exons encoding a part of the extracellular domain determines the ligand specificity of the receptors [11], [12], [13], [14]. The most extraordinary examples of the mutually exclusive exons are in the *Drosophila Dscam* gene [9], [10], which has four clusters of mutually exclusive exons. Selection of only one exon out of 48 candidate exons at a time for the exon 6 cluster is considered to be regulated by a complex system of competing RNA structures and a globally-acting cluster-specific splicing repressor [15], [16]. However, the molecular mechanisms governing the selection patterns for the entire *Dscam* mRNA remain poorly understood [10].

A nematode *Caenorhabditis elegans* is intron-rich like vertebrates and is an excellent model organism for studying the regulation mechanisms of pre-mRNA processing *in vivo* [17]. Up to 25% of its protein-coding genes are estimated to undergo alternative pre-mRNA processing and hundreds of the events are developmentally regulated [18]. We developed a fluorescence alternative splicing reporter system and visualized spatio-temporal selection patterns of mutually exclusive exons in living worms [19], [20], [21]. Through genetic and biochemical analyses, we successfully identified evolutionarily-conserved and broadly-expressed RBFOX (named after RNA binding protein, *fox-1* homolog (*C. elegans*)) family splicing regulators ASD-1 and FOX-1 and a muscle-specific RNA-binding protein SUP-12 as the co-regulators of the muscle-specific selection of exon 5B of the *egl-15* gene encoding the sole homolog of the FGFRs in *C. elegans* [19], [22].

**Figure 1:**
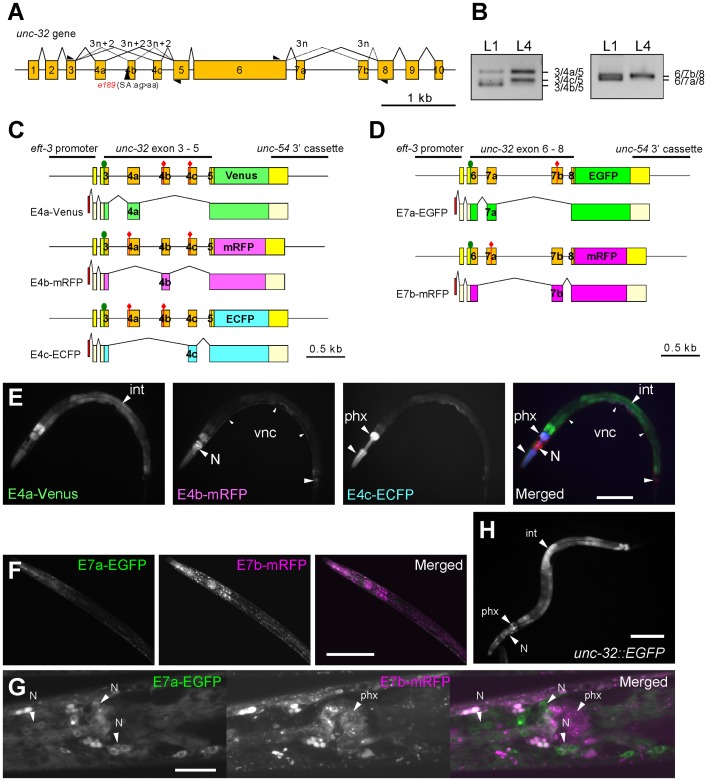
The mutually exclusive exons of the *unc-32* gene are regulated in tissue-specific manners. (*A*) Schematic structure of the *unc-32* gene. Numbered boxes indicate exons. The position of the 3′-splice site mutation in the *unc-32 (e189)* allele is indicated. Black triangles indicate positions and directions of the exonic primers used in the RT-PCR analyses shown in (*B*). (*B*) RT-PCR analyses of *unc-32* exon 4 (left) and exon 7 (right) at the L1 and L4 larval stages. (*C*, *D*) Schematic illustration of the symmetric trio of the exon 4 reporter minigenes (*C*) and the symmetric pair of the exon 7 reporter minigenes (*D*) and the mRNA isoforms derived from them. The cDNA cassettes and the predicted ORFs for Venus/EGFP, mRFP and ECFP are colored in green, magenta and cyan, respectively. Green circles and red diamonds indicate the artificially-introduced initiation and termination codons, respectively. (*E*) Fluorescence images of an L4 larva of the exon 4 reporter worm *ybIs1891 [eft-3::unc-32E4a-Venus eft-3::unc-32E4b-mRFP eft-3::unc-32E4c-ECFP]*. E4a-Venus, E4b-mRFP and E4c-ECFP images in black-white and a merged image with pseudo colors. (*F*, *G*) Confocal images of the exon 7 reporter worms *ybEx1440 [eft-3::unc-32E7-EGFP eft-3::unc-32E7b-mRFP]*. (*H*) A fluorescence image of the *unc-32* transcriptional fusion reporter worm *ybEx1896 [unc-32::EGFP]* containing 1.7-kb of the *unc-32* promoter. int, intestine; N, neurons in head ganglia; phx, pharynx. Scale bars in (*E*), (*F*) and (*H*), 100 µm; in (*G*), 20 µm.

The *unc-32* gene of *C. elegans*, analyzed in this study, encodes the *a* subunit of V_0_ complex of vacuolar-type H^+^-ATPases considered to be proton pumps that acidify intracellular organelles [23], [24]. The unique property of the *unc-32* gene as a model for studying alternative splicing regulation is that it has two sets of mutually exclusive exons (Figure 1A). Only one exon at a time is selected from three exons 4a, 4b and 4c; only one exon is selected at a time from two exons 7a and 7b. Of the six possible combinations of exons 4 and 7, the three isoforms UNC-32A (4a/7b), UNC-32B (4b/7a) and UNC-32C (4c/7b) were predominantly detected [25] and appear to be developmentally regulated [18], raising questions about the exact selection patterns and the regulation mechanisms *in vivo*. In the present study, we demonstrate that *unc-32* exon 4 and exon 7 are selected in tissue-specific manners and that a neuron-specific RNA-binding protein UNC-75 regulates the neuron-specific selection of exons 4b and 7a.

## Results

### Fluorescence splicing reporters for *unc-32* mutually exclusive exons exhibited tissue-specific patterns

We first confirmed mutually exclusive selection of endogenous *unc-32* exon 4 and exon 7 by RT-PCR (Figure 1B). Consistent with the previous report based on microarray profiling and high-throughput sequencing of mRNAs from synchronized worms [18], the splicing patterns of both exon 4 and exon 7 appeared to be developmentally regulated; the relative amounts of the exon 4b isoform and the exon 7a isoform dramatically decreased at the L4 stage (Figure 1B).

Next we visualized the selection patterns of *unc-32* exon 4 and exon 7 *in vivo* by applying our fluorescence alternative splicing reporter system [20]. A trio of symmetric reporter minigenes for exon 4 was constructed by cloning the genomic fragment spanning from exon 3 through exon 5 upstream of one of three fluorescent protein cDNA cassettes and by introducing artificial termination codons into two of the three mutually exclusive exons in each construct (Figure 1C). From these minigenes, we expect expression of Venus-fusion protein (E4a-Venus), monomeric red fluorescent protein (mRFP)-fusion protein (E4b-mRFP) and enhanced cyan fluorescent protein (ECFP)-fusion protein (E4c-ECFP) only when exon 4a alone, 4b alone and 4c alone are selected, respectively (Figure 1C). In the same way, a pair of symmetric exon 7 reporter minigenes was constructed by cloning the genomic fragment spanning from exon 6 through exon 8 upstream of either of two fluorescent protein cDNA cassettes and by introducing an artificial termination codon into one of the two mutually exclusive exons in each construct (Figure 1D). From these minigenes, we expect expression of enhanced green fluorescent protein (EGFP)-fusion protein (E7a-EGFP) and mRFP-fusion protein (E7b-mRFP) when exon 7a and exon 7b are selected, respectively (Figure 1D).

We utilized a ubiquitous promoter to drive expression of the minigenes and generated transgenic reporter worms (Figure 1E–1G). Expression of the three fluorescent proteins in the exon 4 reporter worms varied among tissues; intestine, the nervous system and pharynx predominantly or exclusively expressed E4a-Venus, E4b-mRFP and E4c-ECFP, respectively (Figure 1E). Expression of the two fluorescent proteins in the exon 7 reporter worms also showed tissue-specificity. Most tissues predominantly expressed E7b-mRFP and therefore the worms appear almost Red (Figure 1F). Confocal microscopy revealed that neurons in head ganglia predominantly expressed E7a-EGFP (Figure 1G). The expression patterns of the exon 4 and exon 7 reporters were consistent throughout development. We suspected that lack of the developmental change in the reporter expression was due to ectopic expression of the reporters in tissues that do not express the endogenous *unc-32* gene. A transcriptional fusion reporter, however, revealed that the *unc-32* promoter drives expression in intestine, neurons and pharynx (Figure 1H), the major tissues where the exon 4 and exon 7 reporters were expressed. We therefore concluded that the mutually exclusive exons of the *unc-32* exon 4 and exon 7 reporter minigenes are selected in tissue-specific and not developmentally regulated manners.

### The RBFOX family and UGCAUG stretch in intron 7b are required for exon 7a selection from the *unc-32* exon 7 reporter in the nervous system

**Figure 2:**
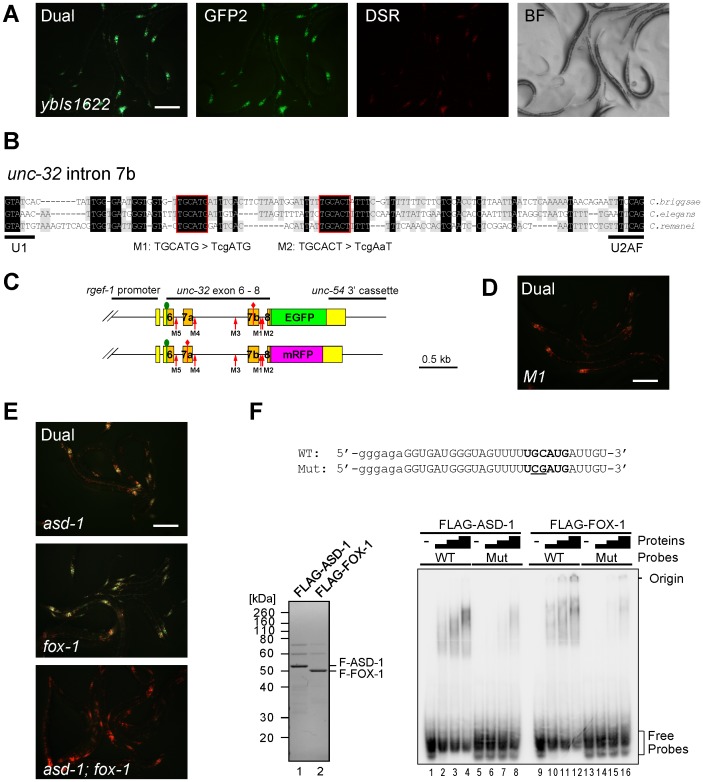
The UGCAUG stretch and the RBFOX family proteins are involved in the neuron-specific selection of exon 7a from the *unc-32* exon 7 reporter. (*A*) Fluorescence images of the exon 7 reporter worms *ybIs1622 [rgef-1::unc-32E7a-EGFP rgef-1::unc-32E7b-mRFP]* with dual-bandpass (Dual), green (GFP2) and red (DSR) filters and a bright field image (BF). Note that individual neurons differentially express E7a-EGFP and E7b-mRFP in a cell-type-specific pattern. (*B*) Nucleotide sequence alignment of intron 7b from *C. briggsae*, *C. elegans* and *C. remanei*. Residues shaded in black and gray are conserved among three and two species, respectively. Conserved stretches are boxed in red and the sequences of the *M1* and *M2* mutant pairs of the reporter minigenes are indicated. (*C*) Schematic illustrations of the mutated pairs of the exon 7 reporter minigenes. Red arrows indicate the positions of the modification. (*D*) A fluorescence image of the *M1* mutant reporter worms with a dual-bandpass filter. (*E*) Fluorescence images of the *ybIs1622* worms in the *asd-1 (yb978)* (top), *fox-1 (e2643)* (middle) and *asd-1; fox-1* (bottom) backgrounds with a dual-bandpass filter. Scale bars, 200 µm in (*A*), (*D*) and (*E*). (*F*) Top, radiolabelled wild-type (WT) and mutant (Mut) intron 7b probes. The substituted bases are underlined. Lowercase indicates the sequence derived from the T7 promoter. Bottom left, Neutral PAGE and CBB staining of the recombinant FLAG-tagged ASD-1 and FOX-1 proteins. Bottom right, EMSA using the WT and Mut probes without (−) or with 4-fold dilution series of FLAG-ASD-1 and -FOX-1.

To focus on the neuron-specific selection of exon 7a, we utilized the *rgef-1* (also known as F25B3.3) promoter to drive pan-neuronal expression of the exon 7 reporter. As expected, transgenic worms with an integrated reporter allele *ybIs1622 [rgef-1::unc-32E7a-EGFP rgef-1::unc-32E7b-mRFP]* predominantly expressed E7a-EGFP in the nervous system and appeared Green with a dual-bandpass filter (Figure 2A). We therefore used the *rgef-1* promoter for further analyses described below.

As *cis*-elements regulating alternative splicing are often evolutionarily conserved in the genus *Caenorhabditis* [19], [21], [22], [26], we first focused on the five stretches in flanking introns of exons 7a and 7b conserved among *C. elegans*, *C. briggsae* and *C. remanei* (Figure 2B, Figure S1A). We constructed five pairs of mutagenized exon 7 reporter minigenes *M1* to *M5* (Figure 2C, Figure S1A) and found that disruption of the UGCAUG stretch in intron 7b (*M1*) changed the color of the exon 7 reporter from Green to Orange (Figure 2D), while disruption of the other stretches had no apparent effect (Figure S1B). We therefore concluded that the UGCAUG stretch in intron 7b is required for the neuron-specific selection of exon 7a.

The UGCAUG stretches are known to be specifically recognized by the RBFOX family splicing regulators in metazoans including *C. elegans* [27]. We have previously reported that the RBFOX family proteins in *C. elegans*, ASD-1 and FOX-1, redundantly repress *egl-15* exon 5B by specifically binding to the UGCAUG stretch in the upstream intron [19]. The *asd-1; fox-1* double mutant is defective in expression of a muscle-specific fibroblast growth factor receptor (FGFR) isoform EGL-15(5A) and shows the egg-laying-defective (Egl-d) phenotype [19]. To test whether ASD-1 and FOX-1 also regulate the neuron-specific selection of *unc-32* exon 7a, we crossed the reporter allele *ybIs1622* with the *asd-1* and *fox-1* mutants. As expected, the reporter worms turned the color from Green to Yellow in the single mutant backgrounds (Figure 2E, top and middle) and to Orange in the double (Figure 2E, bottom), confirming that ASD-1 and FOX-1 are redundantly involved in the neuron-specific selection of exon 7a from the exon 7 reporter.

To confirm direct and specific binding of ASD-1 and FOX-1 to the UGCAUG stretch in intron 7b *in vitro*, we performed an electrophoretic mobility shift assay (EMSA) using the radiolabelled RNA probes with an intact (WT) and a mutagenized (M1) sequence as in the reporters (Figure 2F, top). Recombinant full-length ASD-1 and FOX-1 proteins (Figure 2F, bottom left) efficiently shifted the mobility of the WT probe (Figure 2F, bottom right, lanes 1–4, 9–12) and less efficiently of the M1 probe (lanes 5–8, 13–16) in a dose-dependent manner, demonstrating direct and specific binding of ASD-1 and FOX-1 to the UGCAUG stretch. These results led to the conclusion that ASD-1 and FOX-1 regulate the selection of exon 7a from the *unc-32* exon 7 reporter via the UGCAUG stretch in intron 7b in the nervous system.

### UNC-75 is required for exon 7a selection from the *unc-32* exon 7 reporter in the nervous system

**Figure 3:**
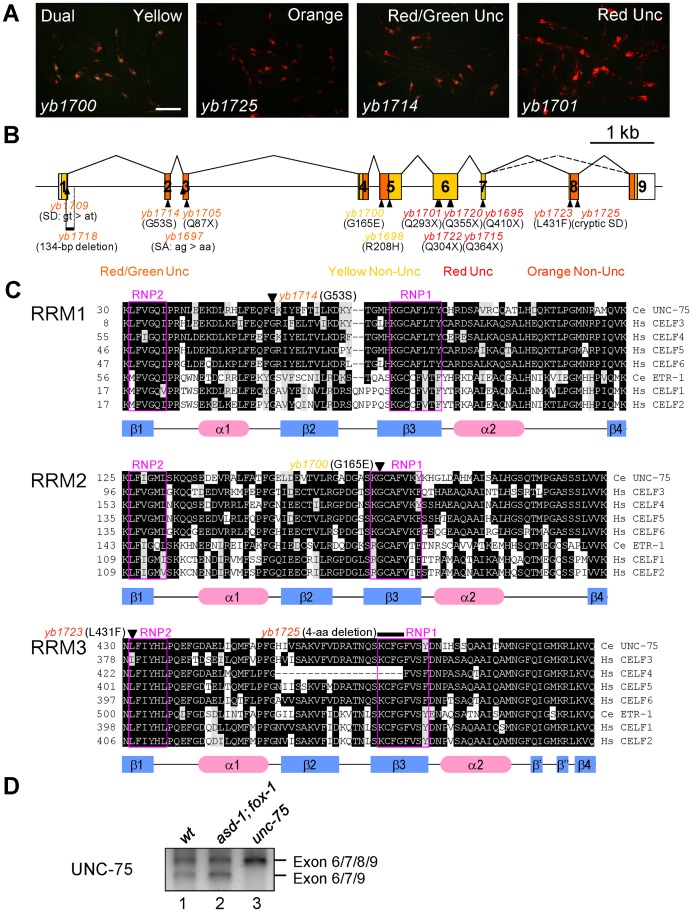
UNC-75 is required for the selection exon 7a from the *unc-32* exon 7 reporter in the nervous system. (*A*) Fluorescence images of the *ybIs1622* worms with the *unc-75* mutant alleles *yb1700*, *yb1725*, *yb1714* and *yb1701* with a dual-bandpass filter. Scale bar, 200 µm. (*B*) Schematic structure of the *unc-75* gene and the positions and consequence of the mutations. The ORF is colored in yellow and the regions corresponding to the three RRMs are in orange. The allele names are in the colors representing the color phenotypes. SA, splice acceptor; SD, splice donor. (*C*) Amino acid sequence alignments of the three RRMs from the CELF3–6 subfamily members *C. elegans* UNC-75, human CELF3, CELF4, CELF5 and CELF6 and the CELF1–2 subfamily members *C. elegans* ETR-1, human CELF1 and CELF2. Conserved residues are shaded in black. Residues with similar properties to the consensus are shaded in gray. The secondary structure elements determined for human CELF1 [51], [52] are depicted with the blue rectangles (β-sheets) and the red ellipses (α-helices) below the alignments. The highly conserved RNP1 and RNP2 motifs in each RRM are boxed in magenta. The positions of the missense mutations and the short deletion are indicated with the allele names. Note that *yb1725* generated a cryptic splice donor site almost exclusively used in the mutant, which resulted in the in-frame deletion of the 4-amino acid stretch indicated. (*D*) RT-PCR analysis of the UNC-75 mRNAs from the synchronized L1 larvae of N2 (*wt*), *asd-1 (yb978); fox-1 (e2643)* and *unc-75 (yb1701)*.

To identify other regulator(s) that confer the neuron-specificity to the exon 7 reporter, we mutagenized the *ybIs1622* strain to screen for mutants exhibiting altered colors. We successfully isolated many homozygous viable strains with Yellow, Orange or Red phenotype (Figure 3A, Figure S2). In some other strains, most neurons turned red while some remained green (Red/Green) (Figure 3A, Figure S2). The color phenotypes were completely penetrated within the strains. Notably, all the Red and Red/Green strains also showed an uncoordinated (Unc) phenotype while the Orange or Yellow strains did not.

By single-nucleotide polymorphism (SNP)-based mapping and sequencing candidate genes, we identified mutations in the *unc-75* gene in the color mutants. The *unc-75* gene was originally identified as the gene responsible for the Unc phenotype caused by defects in synaptic transmission [28]. The exon 7 reporter allele *ybIs1622* crossed with an existing null allele *unc-75 (e950)*, which lacks exon 1 through exon 5 and exhibits the Unc phenotype [28], showed the RedUnc phenotype (data not shown), confirming that the color phenotype is caused by loss of function of the *unc-75* gene.

UNC-75 belongs to the CUG-BP and ETR-3-like factor (CELF) family of RNA-binding proteins, which have two N-terminal RNA recognition motifs (RRMs) followed by a so-called divergent domain and the third RRM at the C-terminus. The CELF family can be divided into two subfamilies CELF1–2 and CELF3–6 according to sequence similarities [29] and UNC-75 is the sole member of the CELF3–6 subfamily in *C. elegans* [29]. Although UNC-75 has been shown to be expressed exclusively in the nervous system and localized to subnuclear speckles [28], it is still unknown what process UNC-75 is involved in.

The mutations identified in the *unc-75* gene are summarized in Figure 3B. All of the five alleles with the RedUnc phenotype have nonsense mutations in exon 6 or exon 7 (Figure 3B). Figure 3C shows amino acid sequence alignments of the three RRMs from the CELF family members in *C. elegans* and human. A missense mutation (*yb1714*) in the conserved glycine residue in the α1β2 loop of RRM1 and four other mutations (*yb1697*, *yb1705*, *yb1709* and *yb1718*) in the region between exon 1 and exon 3 were associated with the Red/GreenUnc phenotype (Figure 3B and 3C, top). A missense mutation (*yb1700*) in the conserved glycine residue in the RNP1 motif of RRM2 (Figure 3B and 3C, middle) and a missense mutation (*yb1698*) in the conserved arginine residue in the divergent domain (Figure S3) were associated with the Yellow phenotype. A missense mutation (*yb1723*) in the RNP2 motif and a 4-aa deletion (*yb1725*) in the RNP1 motif in RRM3 were associated with the Orange phenotype (Figure 3B and 3C, bottom). These results suggested that all the three RRMs and the divergent domain are required for UNC-75 to properly regulate the selection of exon 7a in the nervous system.

During the course of cDNA cloning, we found another UNC-75 mRNA isoform lacking exon 8 corresponding to the anterior half of RRM3 (Figure 3B and 3D, lane 1). Although the skipping of exon 8 does not cause a frame-shift or nonsense-mediated mRNA decay (NMD), the deletion of the half of RRM3 would more significantly affect the function of UNC-75 than the *yb1723* and *yb1725* mutations (Figure 3C, bottom). As many splicing factors are known to regulate their own expression at the pre-mRNA splicing level, we analyzed the effect of the nonsense mutation in the *unc-75* gene on its own mRNAs. The splicing patterns of the UNC-75 mRNAs were not affected in the *asd-1; fox-1* mutant (Figure 3D, lane 2), while the Δexon 8 isoform was undetected in the *unc-75 (yb1701)* mutant (lane 3), consistent with the idea that UNC-75 negatively regulates its own expression by repressing exon 8.

### The C-termini of ASD-1, FOX-1, and UNC-75 function as the sole nuclear localization signals

**Figure 4:**
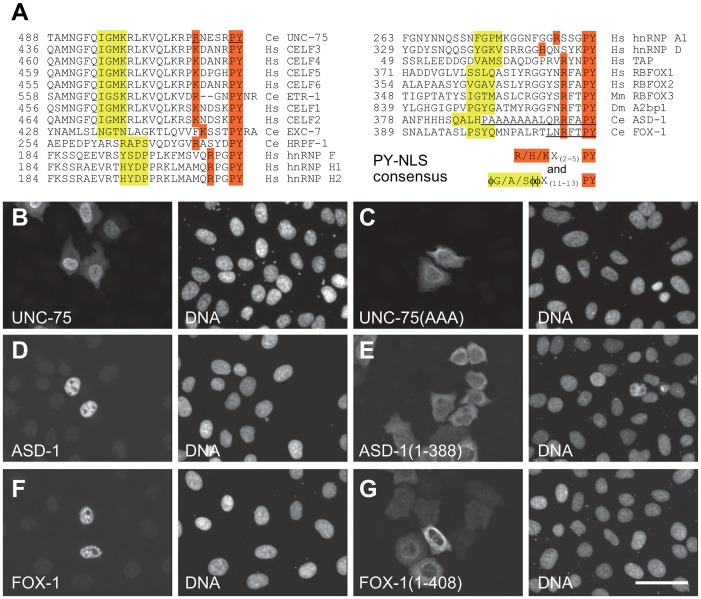
The C-termini of UNC-75, ASD-1, and FOX-1 are the evolutionarily conserved nuclear localization signals. (*A*) Amino acid sequence alignment of the C-terminal ends of the CELF family proteins and the putative PY-NLSs from the related proteins *C. elegans* EXC-7, HRPF-1, human hnRNP F, hnRNP H1 and hnRNP H2 (left), human hnRNP A1, hnRNP D and TAP and the C-terminal ends of the RBFOX family proteins human RBFOX1, RBFOX2, mouse RBFOX3, *Drosophila* A2bp1, *C. elegans* ASD-1 and FOX-1 (right). The consensus sequences of the PY-NLS are indicated. The amino acid residues that match the consensus are shaded in yellow or orange. φ, hydrophobic residues. The underlines in the UNC-75 sequence indicate residues substituted with alanine in UNC-75(AAA) used in (*C*). The underlines in the ASD-1 and FOX-1 sequences indicate residues deleted in the truncated constructs used in (*E*) and (*G*), respectively. (*B*, *C*) Fluorescence images of the HeLa cells transfected with full-length UNC-75 (*B*) and UNC-75(AAA) (*C*) stained with anti-UNC-75 (left panels) and Hoechst 33258 (right panels). (*D*–*G*) Confocal images of the HeLa cells transfected with HA-tagged full-length ASD-1 (*D*), ASD-1(1–388) (*E*), full-length FOX-1 (*F*) and FOX-1(1–408) (*G*) stained with anti-ASD-1 (*D* and *E*, left panels) or anti-FOX-1 (*F* and *G*, left panels) and TO-PRO3 (right panels). Scale bar, 50 µm.

We noticed that the C-termini of the CELF family proteins as well as the RBFOX family proteins are evolutionarily conserved and match the consensus of the hydrophobic PY nuclear localization signal (PY-NLS) [30] (Figure 4A). To test this idea, we analyzed the effect of substitution or deletion of the C-terminal motifs upon subcellular localization of the proteins (Figure 4B–4G). The substitution of the three residues in the PY element of UNC-75 (Figure 4A) disrupted the nuclear localization of UNC-75 (Figure 4B, 4C), confirming that the C-terminal motif of UNC-75 functions as the PY-NLS. In the same way, the deletion of the 7 and 16 residues from the C-termini of ASD-1 and FOX-1, respectively (Figure 4A), disrupted the nuclear localization of the proteins (Figure 4D–4G), indicating that the C-terminal portions of ASD-1 and FOX-1 are the sole NLSs.

### UNC-75 directly and specifically binds to a short fragment in *unc-32* intron 7a *in vitro*

**Figure 5:**
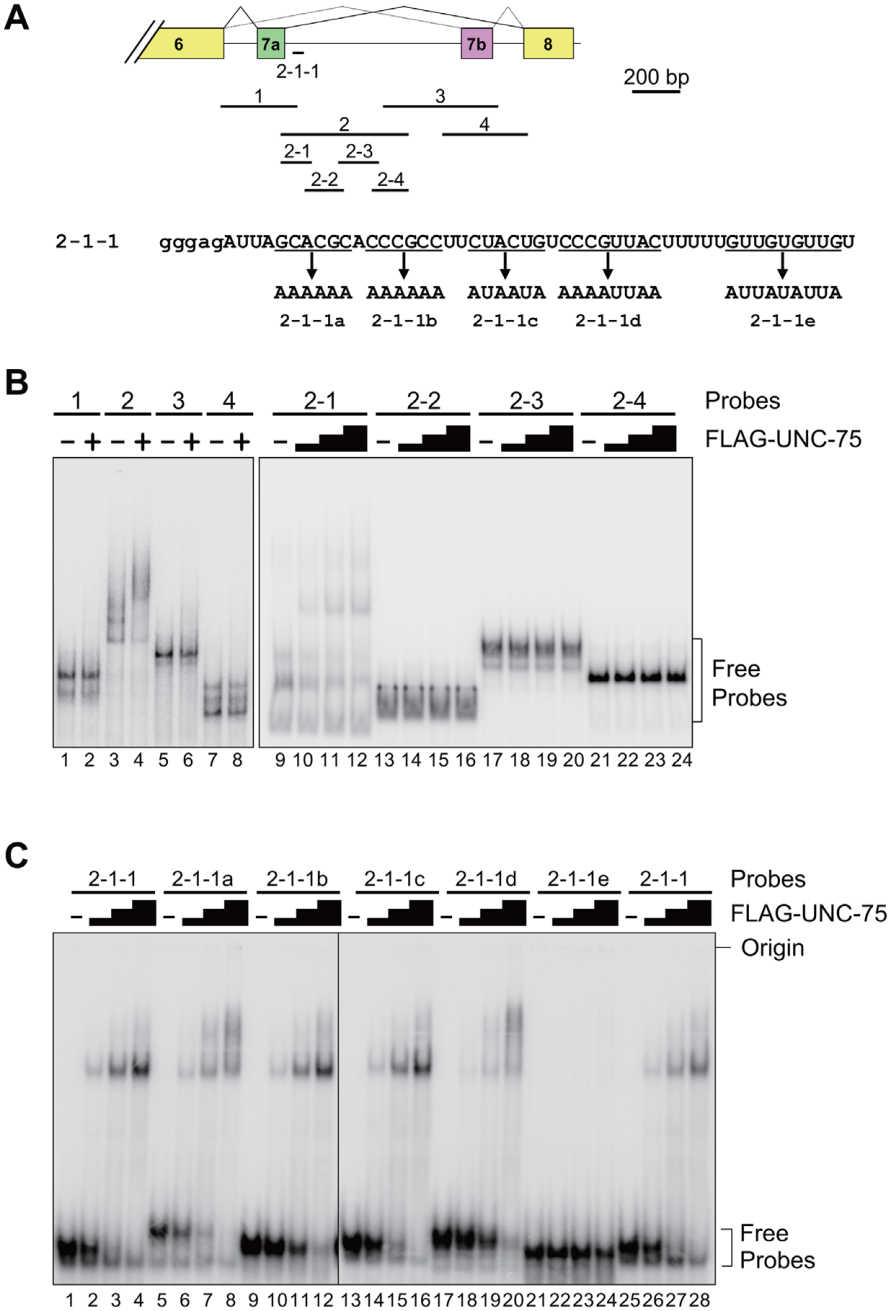
UNC-75 directly and specifically binds to a short fragment in *unc-32* intron 7a *in vitro*. (*A*) Top, schematic illustration of the radiolabelled RNA probes 1 to 4, 2-1 to -4 and 2-1-1 used in the EMSAs. Bottom, sequences of Probe 2-1-1 and its mutants 2-1-1a to -1e. The modified stretches are underlined and the mutant sequences are indicated. Lowercase indicates the sequence derived from the T7 promoter. (*B*) Left, EMSA using the probes 1 to 4 without (−) or with (+) FLAG-tagged recombinant UNC-75 (FLAG-UNC-75). Right, EMSA using the probes 2-1 to 2-4 without (−) or with 2-fold dilution series of FLAG-UNC-75. (*C*) EMSAs using Probe 2-1-1 and its mutants shown in (*A*) without (−) or with 4-fold dilution series of FLAG-UNC-75.

To determine the element(s) in the exon 7 cluster region that UNC-75 directly and specifically recognizes *in vitro*, we performed EMSAs with the radiolabelled RNA probes schematically illustrated in Figure 5A (top panel). Recombinant full-length UNC-75 protein shifted the mobility of Probe 2 (Figure 5B, lanes 3,4) and Probe 2-1 (lanes 9–12) and not of the other probes (Figure 5B). As more than half of Probe 2-1 overlapped with Probe 1 or Probe 2-2, we prepared a shorter probe 2-1-1 (Figure 5A) containing most of the sequence unique to Probe 2-1. UNC-75 shifted the mobility of Probe 2-2-1 (Figure 5C, lanes 1–4, 25–28), demonstrating that UNC-75 directly and specifically binds to the 2-1-1 fragment in this region.

To further specify the element(s) necessary for the UNC-75-binding, we prepared the five mutant probes 2-1-1a to -1e, in each of which G and C residues in a short stretch were replaced with A (Figure 5A, bottom panel). UNC-75 shifted the mobility of the probes 2-1-1a to -1d (lanes 5–20) similarly to Probe 2-1-1, while the mobility of Probe 2-1-1e was unaffected by UNC-75 (lane 21–24), indicating that the UUGUUGUGUUGU stretch disrupted in Probe 2-1-1e is essential for UNC-75 to specifically recognize the 2-1-1 fragment.

### RRM3 of UNC-75 mediates the specific binding to the UUGUUGUGUUGU stretch in *unc-32* intron 7a

**Figure 6:**
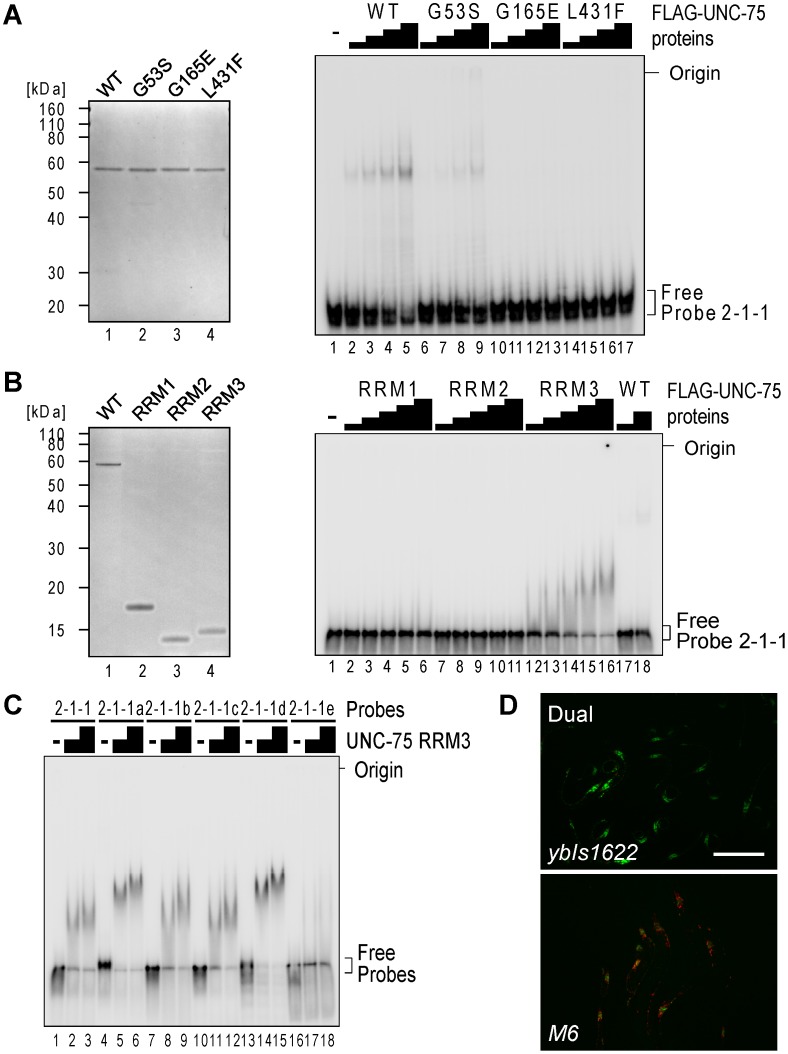
RRM3 of UNC-75 mediates specific binding to the UUGUUGUGUUGU stretch in *unc-32* intron 7a. (*A*) Left, neutral PAGE and CBB staining of the recombinant FLAG-tagged wild-type (WT) UNC-75 protein and the mutant proteins UNC-75(G53S), UNC-75(G165E) and UNC-75(L431F). Right, EMSA using Probe 2-1-1 without or with 2-fold dilution series of the wild-type and mutant UNC-75 proteins. (*B*) Left, CBB staining of the recombinant FLAG-tagged full-length UNC-75 protein (WT; lane 1) and UNC-75(1–114) (RRM1; lane 2), UNC-75(121–213) (RRM2; lane 3) and UNC-75(417–514) (RRM3; lane 4) proteins. Right, EMSA using Probe 2-1-1 and 2-fold dilution series of the three UNC-75 RRM proteins and the full-length protein. (*C*) EMSA using Probe 2-2-1 (lanes 1–3) and five mutant probes (lanes 4–18) without (−) or with 2-fold dilution series of UNC-75 (417–514) protein. (*D*) Fluorescence images of the wild-type (*ybIs1622*; top) and *M6* mutant (bottom) of the exon 7 reporter worms with a dual-bandpass filter. Scale bar, 200 µm.

To test whether all the three RRMs of UNC-75 are involved in the recognition of the 2-2-1 fragment, we performed EMSAs using three mutant recombinant proteins UNC-75 (G53S), UNC-75 (G165E) and UNC-75 (L431F) (Figure 6A, left), each of which had a single missense mutation in one of the three RRMs as found in the mutant alleles. UNC-75 (G53S) and UNC-75 (G165E) less efficiently shifted the mobility of Probe 2-1 and Probe 2-1-1 than wild-type UNC-75 (Figure S4, lanes 1–10; Figure 6A, right, lanes 1–13). UNC-75 (L431F) failed to shift the mobility of these probes (Figure S4, lanes 11–13; Figure 6A, right, lanes 14–17). These results indicated that the missense mutations affected the RNA-binding properties of UNC-75 *in vitro* and that all the three RRMs of UNC-75 are required for the specific recognition of *unc-32* intron 7a.

To specify which of the three RRMs of UNC-75 mediates the specific recognition of the elements in Probe 2-1-1, we prepared recombinant proteins for each of the three RRMs and performed an EMSA (Figure 6B). The RRM3 protein (Figure 6B, right, lanes 12–16) as well as full-length UNC-75 (lanes 17,18) shifted the mobility of Probe 2-1-1, while the RRM1 or RRM2 protein did not (lanes 1-11), indicating that only RRM3 can bind to Probe 2-1-1 by itself. So we used only RRM3 protein for a further EMSA with the mutant 2-1-1 probes. The RRM3 protein shifted the mobility of Probe 2-2-1 (Figure 6C, lanes 1–3) and the mutant probes 2-2-1a to -1d (lanes 4–15) and not of Probe 2-2-1e (lanes 16–18), indicating that RRM3 specifically recognizes the UUGUUGUGUUGU stretch.

To test the requirement of the UUGUUGUGUUGU stretch for the splicing regulation *in vivo*, we constructed another mutant pair of the exon 7 reporter minigenes *M6* that has the same substitutions as in Probe 2-2-1e and generated transgenic worms. The disruption of the UUGUUGUGUUGU stretch turned the color into Orange (Figure 6D), confirming that the stretch is essential for the selection of exon 7a in the nervous system *in vivo*.

### The RBFOX family and UNC-75 differentially regulate the exon 7a selection

**Figure 7:**
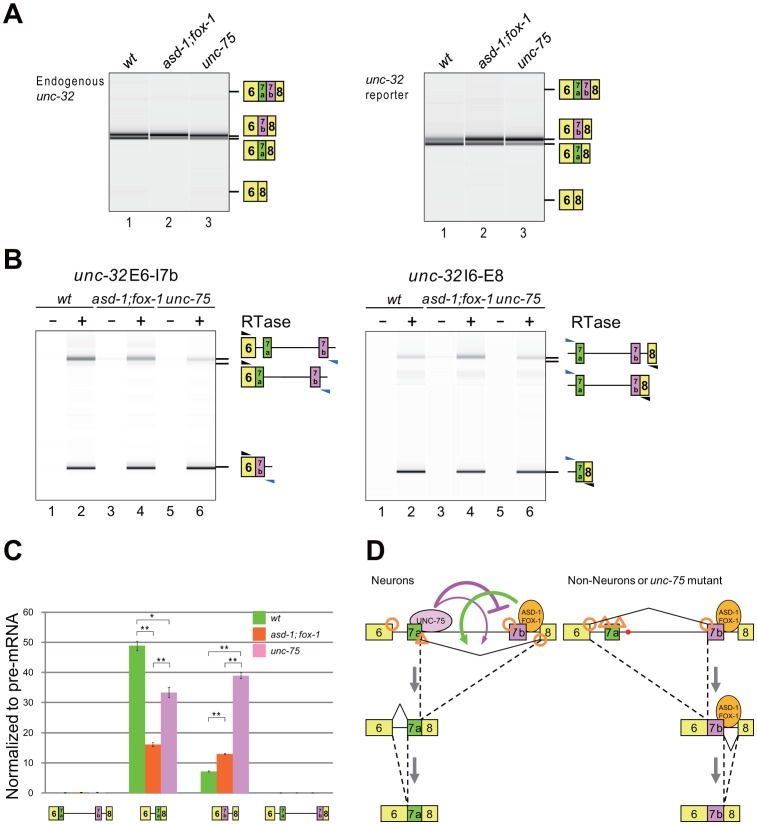
The RBFOX family and UNC-75 differentially regulate the intron excision from the *unc-32* pre-mRNA. (*A*) RT-PCR analysis of the mature mRNAs from the endogenous *unc-32* gene (left) and the exon 7 reporter transgene *ybIs1622* (right) in the wild-type (lane 1), *asd-1 (yb978); fox-1 (e2643)* double (lane 2) and *unc-75 (yb1725)* (lane 3) backgrounds. Note that the isoform with double inclusion or double skipping of exons 7a and 7b was not detected. (*B*) RT-PCR analyses of the partially spliced RNAs from *ybIs1622* in the wild-type, *asd-1; fox-1* and *unc-75* backgrounds. The schematic structures of the RT-PCR products are indicated on the right. Black and blue arrowheads indicate the positions and directions of the exonic and the intronic primers, respectively. RTase, reverse transcriptase. (*C*) Averages of the relative amounts of the partially spliced RNAs to the pre-mRNA. Error bars indicate S.E.M. (n = 3). *p<0.005 and **p<0.001, Student's t-test. (*D*) Schematic models of the mutually exclusive selection of *unc-32* exons 7a and 7b. See Discussion for detail.

Next we analyzed the effects of the RBFOX family and UNC-75 on the endogenous *unc-32* gene. In the wild-type L1 larvae, the exon 7a and exon 7b mRNA isoforms were almost equally detected (Figure 7A, left, lane 1). The relative amount of the exon 7a isoform was reduced in the *asd-1; fox-1* double mutant (lane 2) and *unc-75* mutant (lane 3) backgrounds. A double inclusion isoform or a double skipping isoform was not detected in either of the mutants. These results are consistent with their color phenotypes and the splicing patterns of the exon 7 reporter expressed in the nervous system (Figure 7A, right) and confirm that the RBFOX family and UNC-75 regulate the mutually exclusive splicing of exons 7a and 7b of the endogenous *unc-32* gene.

For mutually exclusive alternative splicing, upstream and downstream flanking introns should be sequentially excised (Figure S5A). To obtain insight into the orders of intron removal for the production of the exon 7a and 7b mRNA isoforms, we analyzed the relative amounts of the four partially spliced RNA species to the unspliced RNA from the exon 7 reporter expressed in the nervous system by RT-PCR using two pairs of an intronic primer and a reporter-specific exonic primer. With one primer set, the partially spliced RNA in which exon 6 was spliced to exon 7b (E6/E7b–E8) was detected but the other partially spliced RNA in which intron 6 was removed (E6/E7a-E7b-E8) was almost undetectable in the wild-type, *asd-1; fox-1* double mutant and *unc-75* mutant worms (Figure 7B, left). With the other primer set, the partially spliced RNA in which exon 7a was spliced to exon 8 (E6–E7a/E8) was detected but the other partially spliced RNA in which intron 7b was removed (E6-E7a-E7b/E8) was almost undetectable in these worms (Figure 7B, right). The relative amounts of the four partially spliced RNAs to the unspliced RNA are summarized in Figure 7C. Of the two partially spliced RNAs that are the putative intermediates for the exon 7a isoform, E6–E7a/E8 was predominantly detected and its relative amount was decreased in the mutants. Of the two partially spliced RNAs that are the putative intermediates for the exon 7b isoform, E6/E7b–E8 was predominantly detected and its relative amount was increased in the mutants. Although these partially spliced RNAs may not necessarily be the processing intermediates but instead dead-end products, the changes in the relative amounts of the partially spliced RNAs are in good correlation with the changes in the amounts of the mature mRNA isoforms in the mutants. These results suggest that E6–E7a/E8 and E6/E7b–E8 are the major processing intermediates for the exon 7a and exon 7b isoforms, respectively. Notably, the mutations in the RBFOX family genes and *unc-75* differentially affected the relative amounts of these partially spliced RNAs, suggesting their differential roles in the alternative splicing regulation of *unc-32* exon 7.

We also analyzed the partially spliced RNAs from the endogenous *unc-32* gene with endogenous RNA-specific pairs of primers. The result revealed consistent but weaker effects of the mutations in the RBFOX family genes and *unc-75* on the partially spliced RNAs (Figure S5B–S5C). Considering that the endogenous *unc-32* gene is expressed not only in the nervous system but also in pharynx and intestine that select exon 7b, this result is consistent with the idea that the RBFOX family and UNC-75 regulate the selection exon 7a from the endogenous *unc-32* gene in the same way as from the reporter in the nervous system. Taking the relative strength of the splice sites in this region (Figure S5D) into account, Figure 7D summarizes the schematic models for the mutually exclusive selection of *unc-32* exon 7, which will be discussed later (see Discussion).

### UNC-75 regulates the neuron-specific selection of *unc-32* exon 4b

**Figure 8:**
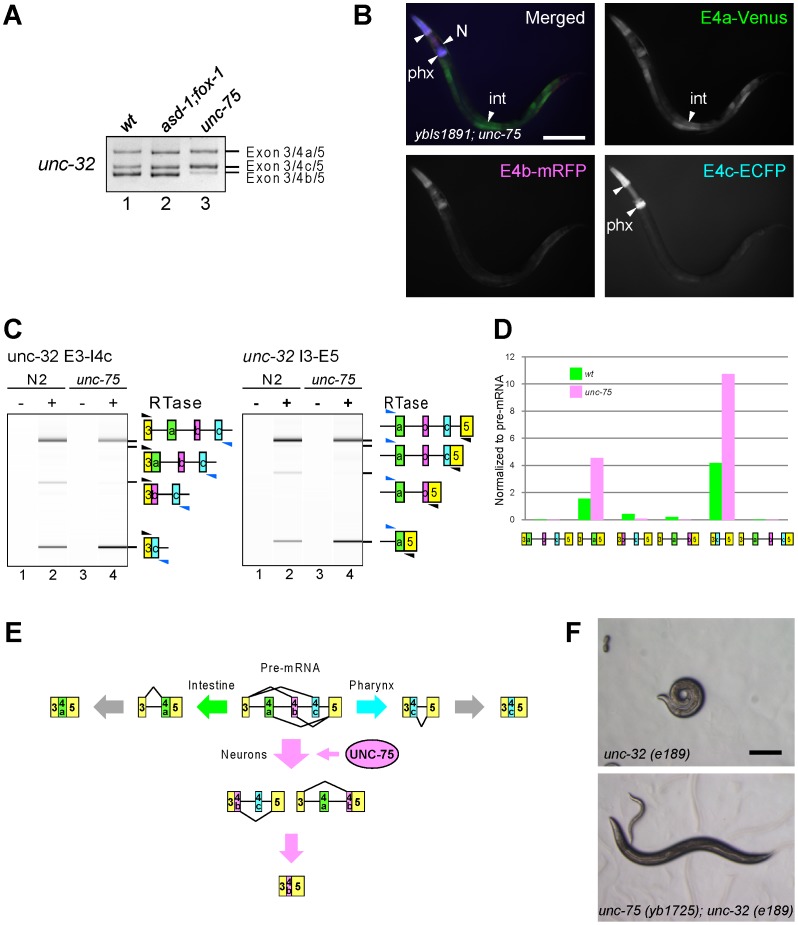
UNC-75 regulates the neuron-specific selection of *unc-32* exon 4b. (*A*) RT-PCR analysis of endogenous *unc-32* exon 4 in the synchronized L1 worms of N2 (*wt*; lane 1), *asd-1 (yb978); fox-1 (e2643)* (lane 2) and *unc-75 (yb1701)* (lane 3). (*B*) Fluorescence images of an L4 worm of the *ybIs1891* reporter allele in the *unc-75 (yb1701)* background as in Figure 1F. Scale bar, 100 µm. (*C*) RT-PCR analyses of the partially spliced RNAs from the endogenous *unc-32* gene in N2 and *unc-75 (yb1701)* as in Figure 7B. (*D*) Relative amounts of the six partially spliced RNAs normalized to the pre-mRNA analyzed in (*C*). (*E*) Schematic illustration of the neuron-specific selection of exon 4b by UNC-75. See Discussion for detail. (*F*) Microphotoimages of the *unc-32 (e189)* (left) and the *unc-75 (yb1725); unc-32 (e189)* (right) worm. Scale bar, 200 µm. Note that the *unc-32* worm exhibits the coiler Unc phenotype.

As *unc-32* exon 4b is also selected in a neuron-specific manner (Figure 1F), we tested whether the RBFOX family and UNC-75 are also involved in the regulation of the exon 4 cluster. Consistent with the absence of a (U)GCAUG stretch in the exon 4 cluster region, the *asd-1; fox-1* double mutation did not affect the splicing patterns of exon 4 of the endogenous *unc-32* gene (Figure 8A, lanes 1, 2). On the other hand, the *unc-75* mutation caused marked reduction of the exon 4b isoform (lane 3). Furthermore, the neuron-specific expression of E4b-mRFP from the exon 4 reporter *ybIs1891* was also abolished in the *unc-75* mutant (Figure 8B, compare with Figure 1F). These results indicated that UNC-75 is required for the selection of exon 4b in the nervous system. We performed an EMSA to localize the UNC-75-binding site(s) with four overlapping probes in the exon 4 cluster region, but none of the probes were shifted as effectively as Probe 2 in Figure 5B by full-length UNC-75 (data not shown). We speculate that other cooperative factors may be required for the specific recognition of the exon 4 cluster region by UNC-75.

We next analyzed the amounts of the six theoretical partially spliced RNAs or putative processing intermediates (Figure S6) from the endogenous *unc-32* gene in the wild type and *unc-75* mutant. Both of the two putative processing intermediate RNAs for the exon 4b isoform were detected in the wild type (Figure 8C, left and right panels, lanes 1, 2) but almost undetectable in the *unc-75* mutant (lanes 3, 4) consistently with the amount of the mature exon 4b isoform. Only one (E3–E4a/E5) of the two partially spliced RNAs that are the putative intermediate RNAs for the exon 4a isoform was detected and its relative amount was increased in the *unc-75* mutant (Figure 8C–8D). Only one (E3/E4c–E5) of the two partially spliced RNAs that are the putative intermediate RNAs for the exon 4c isoform was detected and its relative amount was increased in the *unc-75* mutant (Figure 8C–8D). These results propose a model schematically illustrated in Figure 8E; UNC-75 represses splicing of exon 3 to exon 4c and exon 4a to exon 5 and promotes splicing of exon 4b to exons 3 and 5.

The exon 4b-specific mutation in the *unc-32 (e189)* allele causes the uncoordinated (Unc) phenotype (Figure 1A) [25] and our results demonstrated that exon 4b is specifically selected in the nervous system in an UNC-75-dependent manner. So we speculated that the mutations in *unc-75* should bypass the requirement of exon 4b in the nervous system. Consistent with this idea, the OrangeNon-Unc allele *unc-75 (yb1725)* suppressed the Unc phenotype of the *unc-32 (e189)* mutant (Figure 8F). As neuron-specific ectopic expression of any of the three major isoforms can rescue *unc-32 (e189)* (Figure S7), we reasoned that *unc-75 (yb1725)* suppressed *unc-32 (e189)* via the ectopic expression of the exon 4a or exon 4c isoform in the nervous system. Thus, UNC-75 is the critical splicing factor for the nervous system to specifically select *unc-32* exon 4b *in vivo*.

## Discussion

### Regulation of the mutually exclusive alternative splicing of the *unc-32* gene

In this study, we demonstrated that the two sets of the mutually exclusive exons of the *unc-32* gene are independently regulated in tissue-specific manners by utilizing the fluorescence alternative splicing reporters. Our study revealed that intestine, neurons and pharynx express the UNC-32A (4a/7b), UNC-32B (4b/7a) and UNC-32C (4c/7b) isoforms, respectively. The expression patterns are consistent with the previous report that these three are the major isoforms and that the translational fusion reporter consisting of the *unc-32* promoter through exon 4b is expressed in the nervous system [25]. The neuron-specific isoforms become relatively less abundant in elder stages in the RT-PCR experiments (Figure 1B) probably due to decrease in the relative population and/or mass of the nervous system. Our study thus demonstrated the importance of carefully analyzing alternative splicing patterns at a single cell resolution *in vivo*.

Figure 7D illustrates the proposed models of the neuron-specific selection of exon 7a. In the non-neuronal tissues, exon 7a is skipped presumably due to its weak splice sites and exon 6 is readily spliced to exon 7b (right panel). In neurons, UNC-75 specifically binds to its *cis*-elements in intron 7a to repress exon 7b and the RBFOX family and UNC-75 activate splicing between exon 7a and exon 8 (left panel). The models may explain why the mutations in *unc-75* exerted more sever effects on the selection of exon 7a in the nervous system than the disruption of the RBFOX family genes; in the absence of UNC-75, exon 7b would be readily spliced to exon 6, where the target exon of the RBFOX family is no longer left (right panel).

Figure 8E illustrates the proposed model of the mutually exclusive selection of *unc-32* exon 4. In neurons, UNC-75 activates splicing both between exon 3 and exon 4b and between exon 4b and exon 5 so that exon 4b alone is selected. In intestine and pharynx, splicing between exon 4a and exon 5 and between exon 3 and exon 4c, respectively, occurs first to determine the fate of the pre-mRNA presumably depending on other tissue-specific factor(s). The proposed order of intron excision for each isoform in this model explains the fidelity of the mutually exclusive selection from the three exons of the *unc-32* exon 4 cluster.

The number of the mutually exclusive exons in a cluster is at most two in mammals. The fidelity of the mutually exclusive splicing relies on steric hindrance due to close proximity of the mutually exclusive exons [31], incompatibility between U2-type and U12-type splice sites [32], splicing regulators that repress one exon and activate the other [12], [33] and/or mRNA surveillance system [34]. We have previously raised regulation models for two genes with mutually exclusive exons in *C. elegans*. In the case of *egl-15*, the RBFOX family and SUP-12 cooperatively repress the splice acceptor of the upstream exon [22]. In the case of *let-2*, ASD-2 activates the splice donor of the downstream exon [21]. In the present study, we demonstrate novel types of regulation; for *unc-32* exons 7a and 7b, UNC-75 and the RBFOX family switch the first splicing from E6/E7b to E7a/E8; for *unc-32* exons 4a, 4b and 4c, UNC-75 activates both the splice acceptor and the donor of exon 4b. It has been recently suggested that the mutually exclusive exons in the *slo-1* gene are regulated in intragenic coordination with downstream alternative splicing events although the splicing patters are not analyzed at a single cell resolution [35]. Thus, the order of intron excision and the modes of regulation for the mutually exclusive exons vary from case to case even in the simple model organism.

### Alternative splicing regulation by the CELF3–6 subfamily protein UNC-75

In this study, we identified the first endogenous alternative splicing events regulated by the CELF3–6 subfamily. A recent splicing-sensitive microarray analysis of the *unc-75* mutant suggested only one affected gene, *lec-3* [36], but the selection patterns of the putative target exons in each tissue *in vivo* are not known yet and the function of UNC-75 in the splicing regulation of the *lec-3* gene are to be experimentally defined. In vertebrates, the CELF1–2 subfamily proteins CELF1 (also known as CUG-BP1) and CELF2 (also known as ETR-3 and CUG-BP2) are broadly expressed, highest in heart, skeletal muscle and brain, and their biological functions and biochemical properties are well characterized [29], [37]. On the other hand, CELF3 to CELF6 are predominantly expressed in the nervous system [38], [39], [40], [41], [42] and have been shown to regulate alternative splicing in heterologous minigene systems [33], [38], [43], [44], [45], [46]. However, the *in vivo* functions and biochemical properties of the CELF3–6 subfamily are less characterized [29] presumably due to their functional redundancy.

We identified the short fragment specifically recognized by UNC-75 in *unc-32* intron 7a and provided the genetic and biochemical evidence that all the three RRMs are required for the recognition and regulation of the *unc-32* pre-mRNA (Figure 3C, Figure 6A). Among them, RRM3 recognizes the UUGUUGUGUUGU stretch in the target element by itself (Figure 6C). On the other hand, the stretches that RRM1 and RRM2 recognize could not be determined, although our data shown in Figure 5 and Figure 6 do not preclude the possibility that RRM1 and/or RRM2 also recognize the UUGUUGUGUUGU stretch. These results suggest that recognition of target RNAs by RRM1 and RRM2 is context-dependent or cooperative, which may explain why it is difficult to determine the precise binding sites or consensus sequences for RRM1 and RRM2. The CELF1–2 subfamily has been shown to bind to a variety of UG-rich and related sequences via the three RRMs in a context-dependent manner [47], [48], [49], [50], [51], [52]. Considering the amino acid sequence similarities between the two subfamilies (Figure 3C), it is reasonable that UNC-75 also recognizes the UG-rich sequences. Collection of the *unc-75* mutant alleles revealed that the conserved stretch in the N-terminal portion of the divergent domain is also involved in the recognition and/or splicing regulation of *unc-32* (Figure S3). This is consistent with the previous reports that the N-terminal portion of the divergent domain of CELF4 is involved in the RNA recognition and/or splicing regulation in minigene contexts [44], [46].

The RedUnc mutant alleles have nonsense mutations in *unc-75* exon 6 or 7 (Figure 3A, 3B), while some other mutants show the Red/Green phenotype (Figure 3A, Figure S2), suggesting cell-type-dependent remaining activity of UNC-75 in such mutants. Paradoxically, most of the Red/Green alleles have nonsense mutations or splice site mutations in exon 1, 2 or 3 (Figure 3B), indicative of fatal effects on the UNC-75 expression. The remaining activity of UNC-75 in certain neurons might derive from the use of alternative promoters in the upstream region or in intron 3 to bypass exons 1–3, although we have not experimentally identified such mRNA isoforms from the *unc-75* gene.

### PY-NLS in the RNA–binding proteins

We demonstrated that the C-termini of all the CELF family and the RBFOX family proteins match the consensus of the PY-NLS and that the C-termini are indeed required for the proper nuclear localization of UNC-75, ASD-1 and FOX-1 (Figure 4). As RRM3 of the CELF family resides at the C-terminus, the PY-NLS is overlapping with RRM3 and is highly conserved. It has been reported that deletion of a C-terminal KRP stretch affected the nuclear localization of UNC-75 in neurons [28], consistent with our finding. In contrast to the PY-NLSs in the RBFOX and CELF families, the PY-NLS was originally identified in the internal portion of hnRNP A1 and other RNA-binding proteins including hnRNP D, TAP, HuR, hnRNP F and hnRNP M [30]. Most of the PY-NLSs predicted in many other proteins are structurally divergent and reside in the internal portion [30]. Evolutionary conservation of the sequences and positions of the PY-NLSs in the RBFOX and CELF families may suggest importance of their positions for the functions of these proteins.

### Cooperative regulation of the tissue-specific alternative splicing by the RBFOX family and other splicing regulators

In this and previous studies, we demonstrated that the broadly-expressed RBFOX family proteins ASD-1 and FOX-1 regulate the neuron- and muscle-specific alternative splicing events in a target-specific manner in combination with the neuron-specific RNA-binding protein UNC-75 and the muscle-specific RNA-binding protein SUP-12 [22], respectively. Similarly, an RBFOX family protein RBFOX2 is expressed in a variety of cell types in mammals, yet it can regulate the epithelium-specific alternative splicing of the *FGFR2* gene in coordination with epithelium-specific splicing factors ESRP1 and ESRP2 [11], [12]. The RBFOX family splicing regulators have only one RNA-binding domain that can specifically recognize the (U)GCAUG stretch in the target pre-mRNAs [27], [53]. Therefore, the presence of the (U)GCAUG stretch in the pre-mRNAs is not the sole determinant of the tissue-specificity but can be considered to offer an opportunity for the combinatorial and context-dependent regulation of alternative splicing. Considering their broad expression, the RBFOX family may regulate alternative splicing with a variety of tissue-specificity in cooperation with other tissue-specific factors in both mammals and *C. elegans*.

## Materials and Methods

### Reporter minigene construction

To construct the *unc-32* exon 7 reporter cassettes, the *unc-32* genomic fragment was cloned upstream of either mRFP1 [54] or EGFP (Clontech) cDNA in the Entry vectors by using In-Fusion system (BD Biosciences) and the artificial termination codons were introduced with QuickChange (Stratagene). The reporter minigenes for *unc-32* exon 4 and the *unc-32* transcriptional fusion were constructed as described previously [20]. The sequences of the primers used in the plasmid construction are available in Table S1.

### Worm culture and microscopy

The worms were cultured following standard methods. Generation of transgenic worms, mutant screening and mapping were performed as described previously [20]. The images of the fluorescence reporter and mutant worms were captured using fluorescence stereoscopes (MZ16FA and M205FA, Leica) equipped with color, cooled CCD cameras (DP71, Olympus and DFC310FX, Leica) or a confocal microscope (FV500, Olympus) and processed with Metamorph (Molecular Devices) or Photoshop (Adobe).

### RT–PCR

The RT-PCRs were performed essentially as described previously for amplifying the mature mRNAs [20] and the partially spliced RNAs [55]. The RT-PCR products were analyzed by standard agarose gel electrophoresis or by using BioAnalyzer (Agilent) and the sequences of the RT-PCR products were confirmed by direct sequencing or cloning and sequencing. The sequences of the primers used in the RT-PCR assays are available in Table S2.

### Amino acid sequence alignment

The amino acid sequences of the proteins used in the alignments were retrieved from the protein sequence databases derived from GenBank and RefSeq. The accession numbers are as follows: human CELF1, NP_006551; CELF2, NP_001020247; CELF3, AAK07474; CELF4, NP_064565; CELF5, NP_068757; CELF6, NP_443072; hnRNP A1, AAH02355; hnRNP D, BAA09525; TAP, AAD20016; hnRNP F, NP_004957; hnRNP H1, NP_005511; hnRNP H2, NP_062543; RBFOX1, Q9NWB1; RBFOX2, NP_001026865; mouse RBFOX3, NP_001034256; *Drosophila* A2bp1, AAQ22527; *C. elegans* UNC-75, AAQ19851; ETR-1, NP_493673; EXC-7, CAA85327; HRPF-1, AAK21490; ASD-1, NP_497841; FOX-1, NP_508446. The amino acid sequences were aligned by Clustal W using Lasergene (DNASTAR).

### Antibodies and immunocytochemistry

The rabbit polyclonal anti-UNC-75 antiserum (9493R2R) was generated by using denatured His-tagged full-length UNC-75 protein as described previously [55]. The rabbit polyclonal anti-ASD-1 (RbD8211) and -FOX-1 (RbD8209) antisera were generated with the mixtures of synthetic peptides TVEKLNDFDYKVAL+C and C+RGVPQPGRIPTSTA for anti-ASD-1 and C+GKVKDDPNSDYDLQ and C+LPSYQMNPALRTLN for anti-FOX-1 by Operon Biotechnologies (Tokyo, Japan). The expression vectors for untagged UNC-75 and HA-tagged ASD-1 and FOX-1 were constructed by using Destination vectors pDEST-cDNA3 and pDEST-ME18S-3HA (H.K.), respectively. HeLa cells were transfected with the expression vectors by utilizing GeneJuice (Novagen). For UNC-75, the cells were stained with anti-UNC-75 (9493R2R), Alexa488-conjugated goat anti-rabbit IgG (Molecular Probes) and DAPI (Vector Laboratories) and fluorescence images were captured with a compound microscope (Eclipse E600, Nikon) and a CCD camera (DP71, Olympus). For ASD-1 and FOX-1, the cells were stained with anti-ASD-1 (RbD8211) or -FOX-1 (RbD8209), Cy3-conjugated goat anti-rabbit IgG (Jackson) and TO-PRO3 (Molecular Probes) and the confocal images were acquired with FV1000 (Olympus).

### Electophoretic mobility shift assay (EMSA)

The expression vectors for FLAG-tagged ASD-1, FOX-1 and UNC-75 proteins were constructed using the primers listed in Table S3 and the recombinant proteins were prepared as previously described [55]. The ^32^P-labelled RNA probes were prepared as described previously [55] using the template oligo DNAs listed in Table S4 and the PCR products amplified with the primes in Table S5. The EMSAs were performed as described previously [55].

## Supporting Information

Figure S1 The conserved stretches in introns 6, 7a and 7b are dispensable for the neuron-specific selection of exon 7a except for the UGCAUG stretch. (*A*) Nucleotide sequence alignment of *unc-32* intron 6 (top) and intron 7a (bottom) from *C. briggsae*, *C. elegans* and *C. remanei*. Residues conserved among three and two species are shaded in black and gray, respectively. Red boxes indicate the conserved stretches. Modified sequences in the *M3* to *M5* mutant pairs of the reporter minigenes are indicated. (*B*) Fluorescence images of the transgenic worms expressing the *M2* to *M5* mutant pairs of the *unc-32* exon 7 reporter minigenes with a dual-bandpass filter. Scale bar, 200 µm.
(PDF)

Figure S2 Fluorescence images of the *unc-75* mutants with various color phenotypes. Color images of a representative allele for each phenotype with a dual-bandpass (left), a green (GFP2) and a red (DSR) filter are shown. Scale bar, 200 µm.
(PDF)

Figure S3 Amino acid sequence alignment of the divergent domains from the CELF family proteins in *C. elegans* and human. The amino acid positions are indicated. An arrowhead indicates the amino acid residue substituted in the Yellow mutant allele *unc-75 (yb1698)*.
(PDF)

Figure S4 An EMSA using *unc-32* Probe 2-1 without (−; lane 1) or with 2-fold dilution series of recombinant FLAG-tagged UNC-75 (WT; lanes 2–4), UNC-75 (G53S) (lanes 5–7), UNC-75 (G165E) (lanes 8–10) and UNC-75 (L431F) (lanes 11–13).
(PDF)

Figure S5 The RBFOX family and UNC-75 differentially regulate alternative splicing of *unc-32* exon 7. (*A*) Schematic representation of the four putative pathways to generate the two mature mRNA isoforms. Boxes indicate exons. The four partially spliced RNAs are encircled with gray lines. Colored arrows indicate putative steps that need to be specifically regulated. (*B*) RT-PCR analyses of the partially spliced RNAs from the endogenous *unc-32* gene in the wild-type (*wt*), *asd-1 (yb978); fox-1 (e2643)* and *unc-75 (yb1725)* backgrounds. Schematic structures of the PCR products are indicated on the right. Black and blue arrows indicate the positions and directions of the exonic and intronic primers, respectively. (*C*) Averages of the relative amounts of the partially spliced RNAs to the pre-mRNA. Error bars indicate S.E.M. (n = 3). *p<0.05 and **p<0.01 (Student's t-test). (*D*) Nucleotide sequence alignments of the 5′ and 3′ splice sites of *unc-32* intron 6, intron 7a and intron 7b from *C. elegans*, *C. briggsae*, *C. remanei* and *C. brenneri*. Schematic structure of the exons and relative strength of the splice sites are indicated above the alignments. The nucleotides different from the consensus are in orange.
(PDF)

Figure S6 Schematic representation of the six putative pathways to generate the three mature mRNA isoforms containing one of three mutually exclusive exons. Boxes indicate exons. The six putative intermediate RNAs are encircled with gray lines. Note that the upstream and downstream introns of the selected exon need to be sequentially excised. Colored arrows indicate putative steps that need to be specifically regulated.
(PDF)

Figure S7 Ectopic expression of any one of the three major UNC-32 isoforms in the nervous system can rescue the Unc phenotype of the *unc-32 (e189)* mutant. Arrowheads indicate non-transgenic *unc-32* adult worms. Arrows indicate transgenic *unc-32* adult worms carrying extrachromosomal arrays to drive expression of UNC-32A (left), UNC-32B (middle) or UNC-32C (right) cDNA in the nervous system under the control of the *rgef-1* promoter. Scale bar, 200 µm.
(PDF)

Table S1 Sequences of the primers used in the *unc-32* reporter construction.
(RTF)

Table S2 Sequences of the primers used to detect the *unc-32* and *unc-75* RNAs in RT-PCR assays.
(RTF)

Table S3 Sequences of the primers used in constructing the UNC-75, ASD-1 and FOX-1 expression vectors.
(RTF)

Table S4 Sequences of the oligo DNAs used in *in vitro* transcription.
(RTF)

Table S5 Sequences of the primers used to prepare the templates for *in vitro* transcription.
(RTF)
