# Supplementary material for: CELF Family RNA–Binding Protein UNC-75 Regulates Two Sets of Mutually Exclusive Exons of the unc-32 Gene in Neuron-Specific Manners in Caenorhabditis elegans
Source: PLoS Genet. 2013 Feb 28;9(2):e1003337. doi: 10.1371/journal.pgen.1003337 (PMC3585155; doi:10.1371/journal.pgen.1003337)
Supplement: Figure S1 — The conserved stretches in introns 6, 7a and 7b are dispensable for the neuron-specific selection of exon 7a except for the UGCAUG stretch. (A) Nucleotide sequence alignment of unc-32 intron 6 (top) and intron 7a (bottom) from C. briggsae, C. elegans and C. remanei. Residues conserved among three and two species are shaded in black and gray, respectively. Red boxes indicate the conserved stretches. Modified sequences in the M3 to M5 mutant pairs of the reporter minigenes are indicated. (B) Fluorescence images of the transgenic worms expressing the M2 to M5 mutant pairs of the unc-32 exon 7 reporter minigenes with a dual-bandpass filter. Scale bar, 200 µm. (PDF) [file pgen.1003337.s001.pdf]

Figure S1.

A

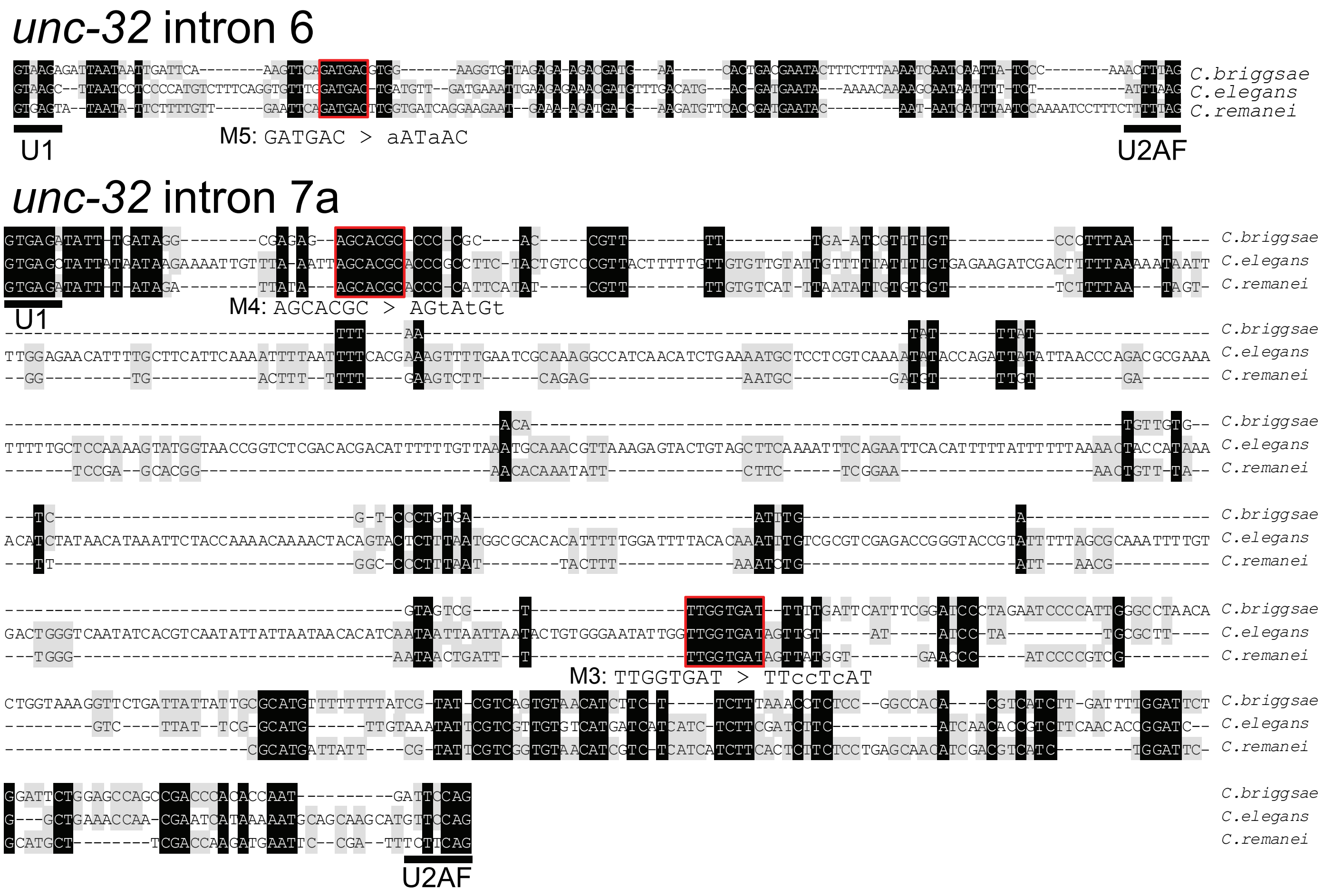
unc-32 intron 6
C.briggsae
C.elegans
C.remanei
U1
M5: GATGAC > aATaAC
U2AF
unc-32 intron 7a
U1
M4: AGCACGC > AGtAtGt
M3: TTGGTGAT > TTccTcAT
U2AF

B

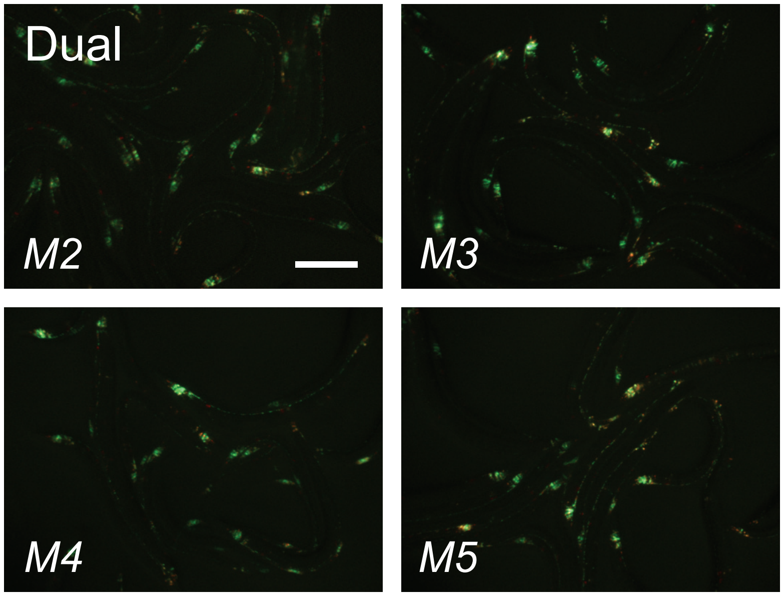
Dual
M2
M3
M4
M5
