# Supplementary figures and images for: CELF Family RNA–Binding Protein UNC-75 Regulates Two Sets of Mutually Exclusive Exons of the unc-32 Gene in Neuron-Specific Manners in Caenorhabditis elegans
Source: PLoS Genet. 2013 Feb 28;9(2):e1003337. doi: 10.1371/journal.pgen.1003337 (PMC3585155; doi:10.1371/journal.pgen.1003337)

Figure S2.

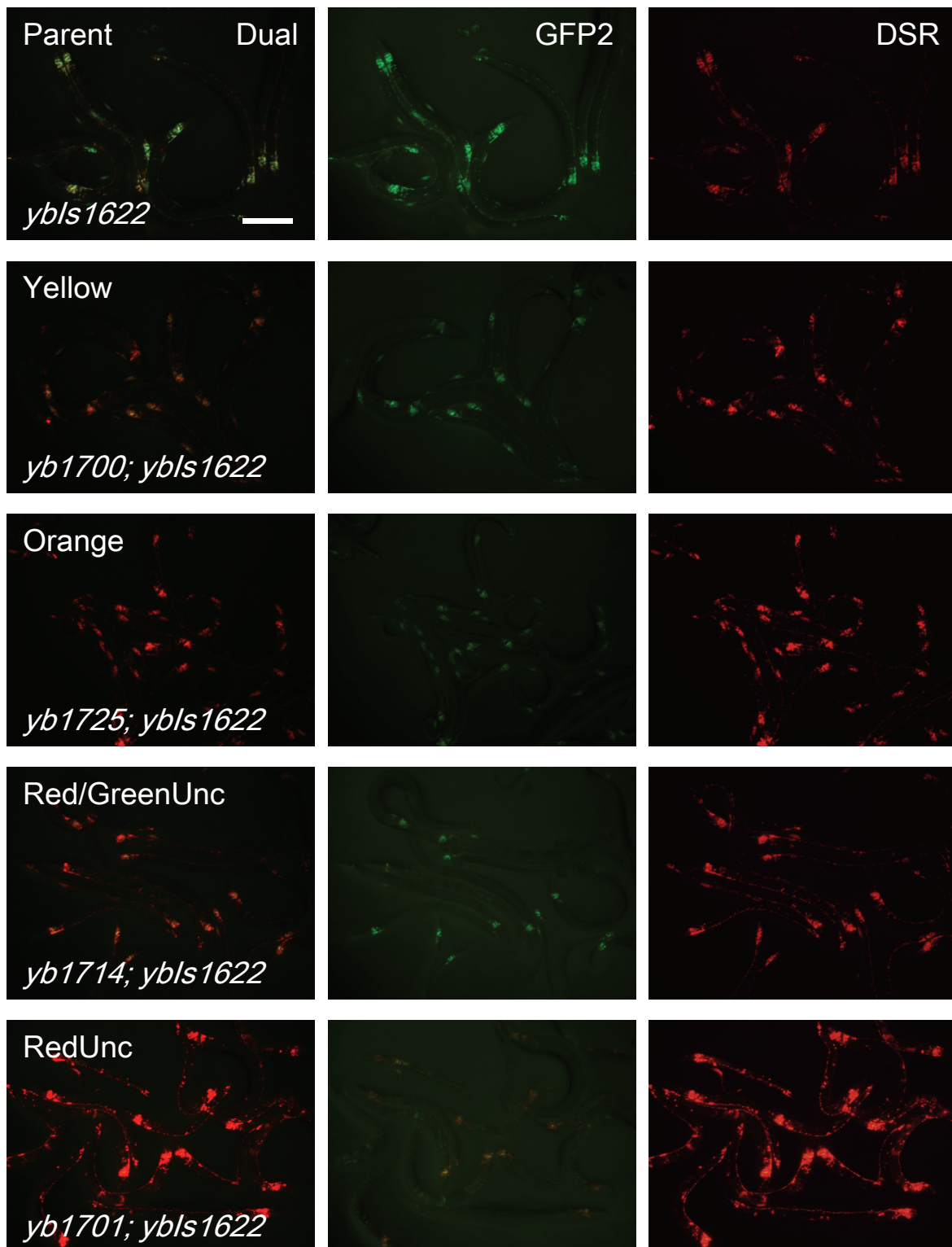

Supplement: Figure S2 — Fluorescence images of the unc-75 mutants with various color phenotypes. Color images of a representative allele for each phenotype with a dual-bandpass (left), a green (GFP2) and a red (DSR) filter are shown. Scale bar, 200 µm. (PDF) [file pgen.1003337.s002.pdf]

Figure S4.

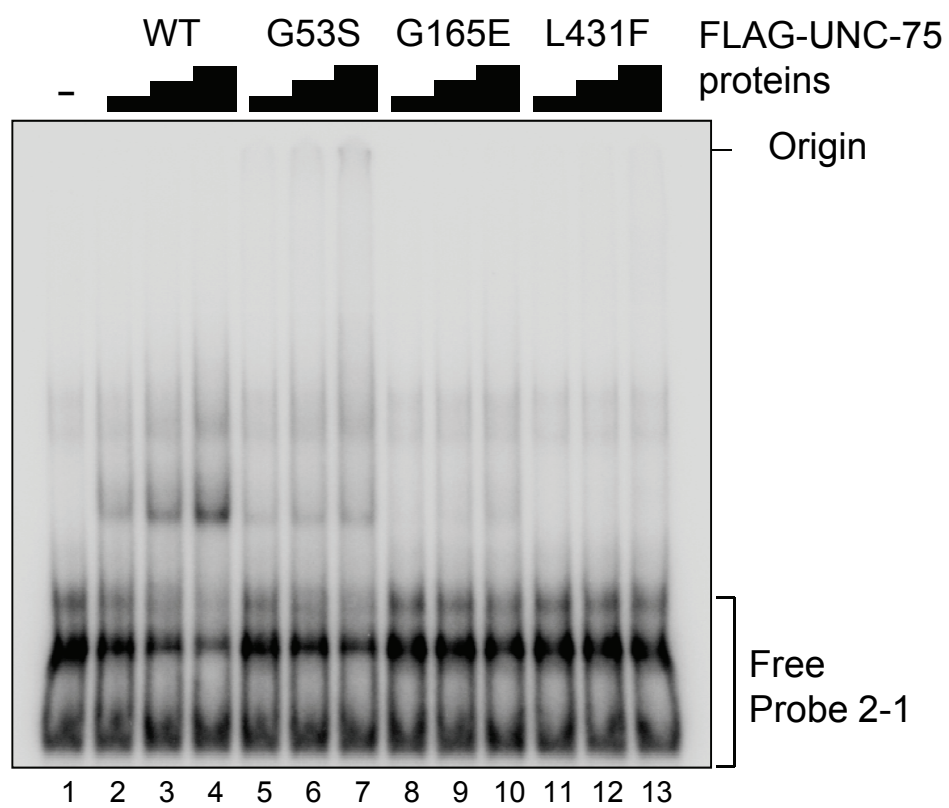

Supplement: Figure S4 — An EMSA using unc-32 Probe 2-1 without (−; lane 1) or with 2-fold dilution series of recombinant FLAG-tagged UNC-75 (WT; lanes 2–4), UNC-75 (G53S) (lanes 5–7), UNC-75 (G165E) (lanes 8–10) and UNC-75 (L431F) (lanes 11–13). (PDF) [file pgen.1003337.s004.pdf]

Figure S6.

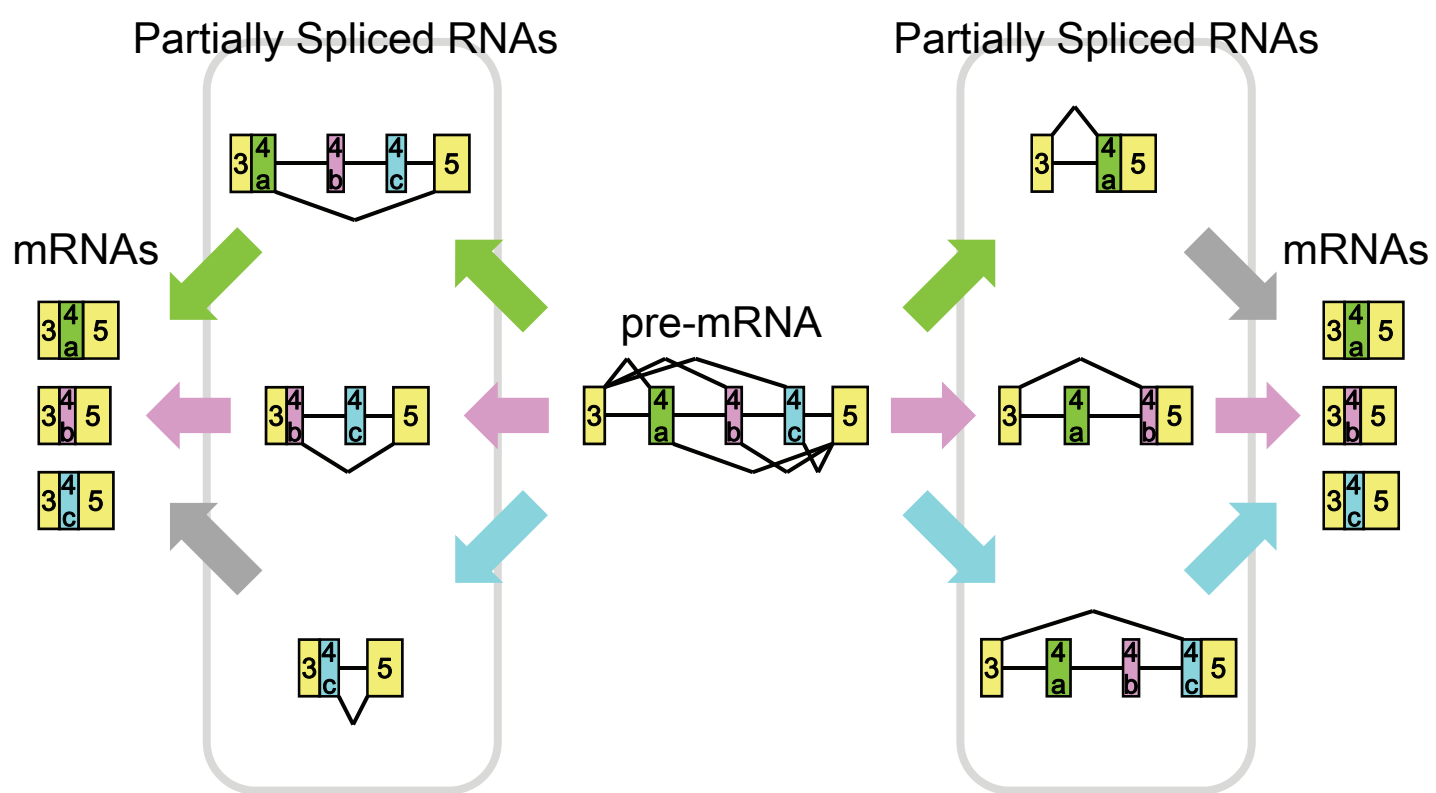

Supplement: Figure S6 — Schematic representation of the six putative pathways to generate the three mature mRNA isoforms containing one of three mutually exclusive exons. Boxes indicate exons. The six putative intermediate RNAs are encircled with gray lines. Note that the upstream and downstream introns of the selected exon need to be sequentially excised. Colored arrows indicate putative steps that need to be specifically regulated. (PDF) [file pgen.1003337.s006.pdf]

Figure S7.

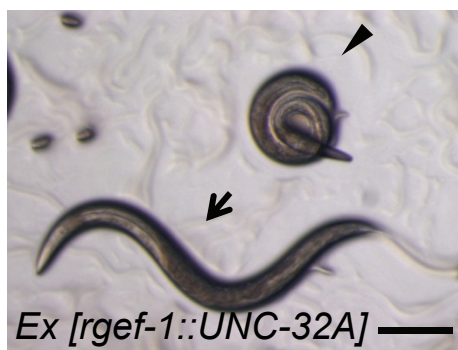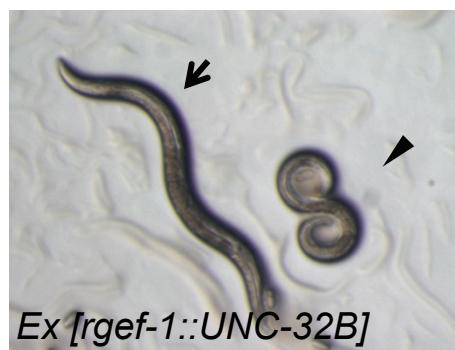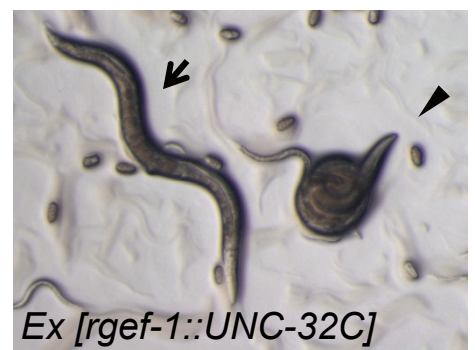

Supplement: Figure S7 — Ectopic expression of any one of the three major UNC-32 isoforms in the nervous system can rescue the Unc phenotype of the unc-32 (e189) mutant. Arrowheads indicate non-transgenic unc-32 adult worms. Arrows indicate transgenic unc-32 adult worms carrying extrachromosomal arrays to drive expression of UNC-32A (left), UNC-32B (middle) or UNC-32C (right) cDNA in the nervous system under the control of the rgef-1 promoter. Scale bar, 200 µm. (PDF) [file pgen.1003337.s007.pdf]
