# Supplementary material for: CELF Family RNA–Binding Protein UNC-75 Regulates Two Sets of Mutually Exclusive Exons of the unc-32 Gene in Neuron-Specific Manners in Caenorhabditis elegans
Source: PLoS Genet. 2013 Feb 28;9(2):e1003337. doi: 10.1371/journal.pgen.1003337 (PMC3585155; doi:10.1371/journal.pgen.1003337)
Supplement: Figure S3 — Amino acid sequence alignment of the divergent domains from the CELF family proteins in C. elegans and human. The amino acid positions are indicated. An arrowhead indicates the amino acid residue substituted in the Yellow mutant allele unc-75 (yb1698). (PDF) [file pgen.1003337.s003.pdf]

Figure S3.

▼ *yb1698* (R208H)

```
201 YADTEKERQNR----------------------RM-QQMAAQMG------MLNPMLV Ce UNC-75
172 FADTEKERGLR----------------------RM-QQVATQLG------MFSP-IA Hs CELF3
229 FADTDKERTMR----------------------RM-QQMAGQMG------MFNP-MA Hs CELF4
211 FADTDKERTLR----------------------RM-QQMVGQLG------ILTPSLT Hs CELF5
211 LADTDRERALR----------------------RM-QQMAGHLG------AFHP-AP Hs CELF6
219 FADTQKDKDVKTKSMITGSGAGSPKGGAASLLQNLNPALLQQLGGGQNYQAVASLLS Ce ETR-1
185 FADTQKDKEQK----------------------RMAQQLQQQMQ------QISAASV Hs CELF1
185 FADTQKDKEQR----------------------RLQQQLAQQMQ------QLNTAT- Hs CELF2

229 NQVGMQYNAYQQVLQQQSLAAQTNAMASAAYLPLLQQQTTDP-LHVLQLQAAAAAAQ Ce UNC-75
199 LQFG-AYSAYTQALMQQQ-AALVA--AHSAYLSPMATMAAVQ-MQ----HMAAINAN Hs CELF3
256 IPFG-AYGAYAQALMQQQ-AALMASVAQGGYLNPMAAFAAAQ-MQ----QMAALNMN Hs CELF4
239 LPFS-PYSAYAQALMQQQ-TTVLST--SGSYLSPGVAFSPCH-IQ----QIGAVSLN Hs CELF5
238 LPLG-ACGAYTTAILQHQ-AALLAA-AQGPGLGPVAAVAA-Q-MQ----HVAAFS-- Hs CELF6
276 LINGQQGQQQQHQQQQNVLGILGTVLSALGKLTEGGDDASAKSSSEKPRHQALMTSP Ce ETR-1
214 WGNLAGLNTLGPQYL-----ALLQQTASSGNLNTLSSLHPMGGLNAMQLQNLAALAA Hs CELF1
213 WGNLTGLGGLTPQYL-----ALLQQATSSSNLGAFSGIQQMAGMNALQLQNLATLAA Hs CELF2

285 AAANPVLSQQTQQQQQQQQL-------------------AAQLQLQSAAAQNPHYAL Ce UNC-75
247 GLIATPITPSSGTSTPPAIAA------------------TPVSAIPAALGVNGYSPV Hs CELF3
306 GLAAAPMTPTSGGSTPPGITA------------------PAVPSIPSPIGVNGFTGL Hs CELF4
287 GLPATPIAPASGLHSPPLLGT------------------TAVPGLVAPIT-NGFAGV Hs CELF5
284 -LVAAPLLPAAAANSPPGSGP------------------GTLPGLPAPIGVNGFGPL Hs CELF6
333 APTATSSTSSSASHHHQQHQQQLSQQQQQQQHPQQQGLGNPLLGNPAMAAQNQFDAI Ce ETR-1
266 AASAAQNTPSGTNA--LTTSS------------------SPLSVLTSS--GSS-PSS Hs CELF1
265 AAAAAQTSATSTNANPLSTTS------------------SALGALTSPVAAST-PNS Hs CELF2

324 AAQALAQQQAAQQAQAVVAHSHAQVHQHQTAQVTSTASHAQTENPAASSYG-SLAAA Ce UNC-75
286 PTQPTGQPAPDALYPNGVHPYPAQSPVAPVDPLQQAYAGMQ-HYT--AAYP--AAYS Hs CELF3
345 PPQANGQPAAEAVFANGIHPYPAQSPTAA-DPLQQAYAGVQ-QYAGPAAYP--AAYG Hs CELF4
325 VPFPGGHPALETVYANGLVPYPAQSPTVA-ETLHPAFSGVQ-QYT--AMYP-TAAIT Hs CELF5
322 TPQTNGQPGSDTLYNNGLSPYPAQSPGVA-DPLQQAYAGMH-HYA--AAYP--SAYA Hs CELF6
390 TMAQIAHQQ-QMLALQGFAV-QQGAPSQQQQGLAGGMAGAKT--TSPVAASLANHQQ Ce ETR-1
300 SSSNSVNPIASLGALQTLAGATAGLN-------VGSLAGMAALNGGLGSSGLSNGTG Hs CELF1
303 TAGAAMNSLTSLGTLQGLAGATVGLNNINALAVAQMLSGMAALNGGLGATGLTNGTA Hs CELF2

380 AAAANYSSLLSGMESQHN-------AAAALQLAQIQQQAAAALPMVTPREVLGPDGC Ce UNC-75
338 LVAPAFPQPPALVAQQPP-------PPPQQQQQQQQQQQQ--------RE--GPDGC Hs CELF3
398 QISQAFPQPPPMIPQQ-----------------------Q--------RE--GPEGC Hs CELF4
377 PIAHSVPQPPPLL-----------------------QQQQ--------RE--GPEGC Hs CELF5
373 PVSTAFPQQPSALPQQ-----------------------Q--------RE--GPEGC Hs CELF6
443 IALTPFAGGAAALDHFQAMQQYALLANLQATGGVGVQATTSAAQMVGNGDVKGPDGA Ce ETR-1
350 STMEALTQAYSGIQQYAA-------AALPTLYNQNLLTQQSIGAAGSQKE--GPEGA Hs CELF1
360 GTMDALTQAYSGIQQYAA-------AALPTLYSQSLLQQQS--AAGSQKE--GPEGA Hs CELF2
```
