# Supplementary material for: CELF Family RNA–Binding Protein UNC-75 Regulates Two Sets of Mutually Exclusive Exons of the unc-32 Gene in Neuron-Specific Manners in Caenorhabditis elegans
Source: PLoS Genet. 2013 Feb 28;9(2):e1003337. doi: 10.1371/journal.pgen.1003337 (PMC3585155; doi:10.1371/journal.pgen.1003337)
Supplement: Figure S5 — The RBFOX family and UNC-75 differentially regulate alternative splicing of unc-32 exon 7. (A) Schematic representation of the four putative pathways to generate the two mature mRNA isoforms. Boxes indicate exons. The four partially spliced RNAs are encircled with gray lines. Colored arrows indicate putative steps that need to be specifically regulated. (B) RT-PCR analyses of the partially spliced RNAs from the endogenous unc-32 gene in the wild-type (wt), asd-1 (yb978); fox-1 (e2643) and unc-75 (yb1725) backgrounds. Schematic structures of the PCR products are indicated on the right. Black and blue arrows indicate the positions and directions of the exonic and intronic primers, respectively. (C) Averages of the relative amounts of the partially spliced RNAs to the pre-mRNA. Error bars indicate S.E.M. (n = 3). *p<0.05 and **p<0.01 (Student's t-test). (D) Nucleotide sequence alignments of the 5′ and 3′ splice sites of unc-32 intron 6, intron 7a and intron 7b from C. elegans, C. briggsae, C. remanei and C. brenneri. Schematic structure of the exons and relative strength of the splice sites are indicated above the alignments. The nucleotides different from the consensus are in orange. (PDF) [file pgen.1003337.s005.pdf]

Figure S5.

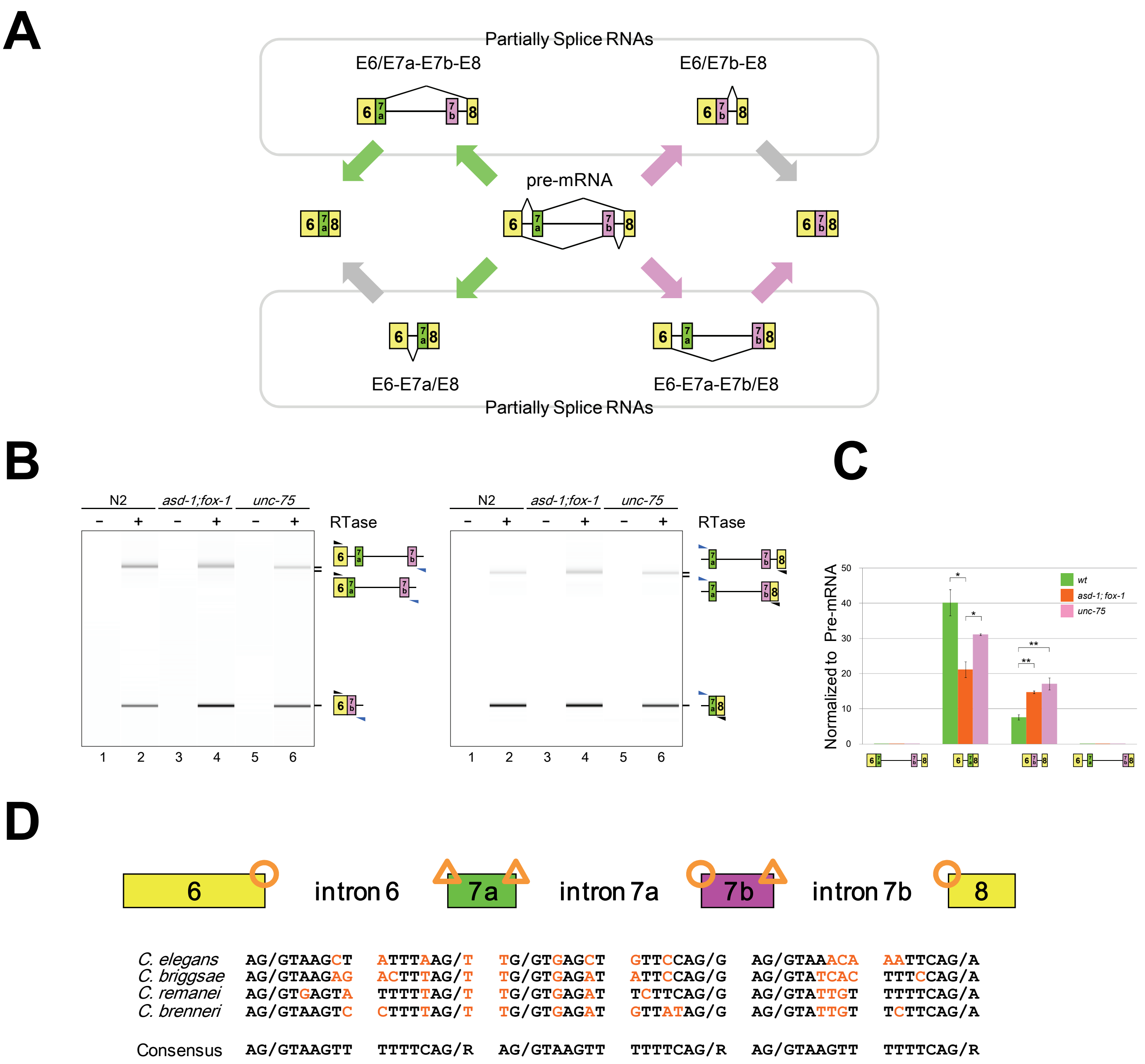

A
Partially Splice RNAs
E6/E7a-E7b-E8
E6/E7b-E8
pre-mRNA
E6-E7a/E8
E6-E7a-E7b/E8
Partially Splice RNAs
B
N2
asd-1;fox-1
unc-75
RTase
1 2 3 4 5 6
C
Normalized to Pre-mRNA
0 10 20 30 40 50
wt
asd-1; fox-1
unc-75
D
6
intron 6
7a
intron 7a
7b
intron 7b
8
C. elegans AG/GTAAGCT ATTTAAG/T TG/GTGAGCT GTTCCAG/G AG/GTAAACA AATTCAG/A
C. briggsae AG/GTAAGAG ACTTTAG/T TG/GTGAGAT ATTCCAG/G AG/GTATCAC TTTCCAG/A
C. remanei AG/GTGAGTA TTTTTAG/T TG/GTGAGAT TCTTCAG/G AG/GTATTGT TTTTCAG/A
C. brenneri AG/GTAAGTC CTTTTAG/T TG/GTGAGAT GTTATAG/G AG/GTATTGT TCTTCAG/A
Consensus AG/GTAAGTT TTTTCAG/R AG/GTAAGTT TTTTCAG/R AG/GTAAGTT TTTTCAG/R
