# Supplementary material for: CELF Family RNA–Binding Protein UNC-75 Regulates Two Sets of Mutually Exclusive Exons of the unc-32 Gene in Neuron-Specific Manners in Caenorhabditis elegans
Source: PLoS Genet. 2013 Feb 28;9(2):e1003337. doi: 10.1371/journal.pgen.1003337 (PMC3585155; doi:10.1371/journal.pgen.1003337)
Supplement: Table S1 — Sequences of the primers used in the unc-32 reporter construction. (RTF) [file pgen.1003337.s008.rtf]

Table S1. Sequences of the primers used in the unc-32 reporter construction. 	
Primers used to amplify the unc-32 genomic fragments	
Sequence	Constructed cassettes and minigenes	
5'-AAAAGCAGGCTCTAGACCATGGTTTCAGAGCTCACAGAGCTG-3'	unc-32E4a-Venus, unc-32E4b-mRFP, unc-32E4c-ECFP	
5'-TATACAAAGTTGGACGTTCCCGTTGAATTACACC-3'	unc-32E4a-Venus, unc-32E4b-mRFP, unc-32E4c-ECFP	
5'-AAAAGCAGGCTCTAGACCATGGTCATGATGAAAAGTCGT-3'	unc-32E7a-EGFP, unc-32E7b-mRFP	
5'-CCATGGTGGCTCTAGATTCTGCGTCATCCTGGTTGAT-3'	unc-32E7a-EGFP, unc-32E7b-mRFP	
Underlines indicate the attB sequences. 	
		
Primers used for the mutagenesis	
Sequence	Constructed cassettes	
5'-TCATGATCGGTGGtaAATTCTGGAAGGC-3'	unc-32E4b-mRFP, unc-32E4c-ECFP	
5'-GCCTTCCAGAATTtaCCACCGATCATGA-3'	unc-32E4b-mRFP, unc-32E4c-ECFP	
5'-GTTGCCACCTGCTtaAaTCGAATCTGAAGA-3'	unc-32E4a-Venus, unc-32E4c-ECFP	
5'-TCTTCAGATTCGAtTtaAGCAGGTGGCAAC-3'	unc-32E4a-Venus, unc-32E4c-ECFP	
5'-TGCCTCATCAGCGtAAAGTTCGGGAAT-3'	unc-32E4a-Venus, unc-32E4b-mRFP	
5'-ATTCCCGAACTTTaCGCTGATGAGGCA-3'	unc-32E4a-Venus, unc-32E4b-mRFP	
5'-CCGTTATGCTATTCtGAAAGCCTTACTTC-3'	unc-32E7b-mRFP	
5'-GAAGTAAGGCTTTCaGAATAGCATAACGG-3'	unc-32E7b-mRFP	
5'-TTGCGAAACCATAaTTTCTGTATCGCC-3'	unc-32E7a-EGFP	
5'-GGCGATACAGAAAtTATGGTTTCGCAA-3'	unc-32E7a-EGFP	
5'-ATGGGTAGTTTTTcgATGATTGTATTAG-3'	unc-32E7a-EGFP-M1, unc-32E7b-mRFP-M1	
5'-CTAATACAATCATcgAAAAACTACCCAT-3'	unc-32E7a-EGFP-M1, unc-32E7b-mRFP-M1	
5'-ATTAGTTTTATTCTcgAaTTTTTCCAATATTA-3'	unc-32E7a-EGFP-M2, unc-32E7b-mRFP-M2	
5'-TAATATTGGAAAAAtTcgAGAATAAAACTAAT-3'	unc-32E7a-EGFP-M2, unc-32E7b-mRFP-M2	
5'-GGGAATATTGGTTccTcATAGTTGTATATC-3'	unc-32E7a-EGFP-M3, unc-32E7b-mRFP-M3	
5'-GATATACAACTATgAggAACCAATATTCCC-3'	unc-32E7a-EGFP-M3, unc-32E7b-mRFP-M3	
5'-TTGTTTAAATTAGtAtGtACCCGCCTTCTAC-3'	unc-32E7a-EGFP-M4, unc-32E7b-mRFP-M4	
5'-GTAGAAGGCGGGTaCaTaCTAATTTAAACAA-3'	unc-32E7a-EGFP-M4, unc-32E7b-mRFP-M4	
5'-TTTCAGGTGTTTGaATaACTGATGTTGATG-3'	unc-32E7a-EGFP-M5, unc-32E7b-mRFP-M5	
5'-CATCAACATCAGTtATtCAAACACCTGAAA-3'	unc-32E7a-EGFP-M5, unc-32E7b-mRFP-M5	
5'-CTGTCCCGTTACTTTTTaTTaTaTTaTATTGTTTTTATTTTGTG-3'	unc-32E7a-EGFP-M6, unc-32E7b-mRFP-M6	
5'-CACAAAATAAAAACAATAtAAtAtAAtAAAAAGTAACGGGACAG-3'	unc-32E7a-EGFP-M6, unc-32E7b-mRFP-M6	
Lowercase indicates the nucleotides different from the wild type. 	
		
Primers used for amplifying the unc-32 promoter	
Sequence	Constructed reporter minigene	
5'-GATAAGCTTCGGAGACTGTATGGTGAGCTGGTG-3'	punc-32p-EGFP	
5'-CGCACTAGTTCACGTAATCACCgATTCTAGTTG-3'	punc-32p-EGFP	
Underlines indicate the restriction enzyme recognition sites used for cloning. Lower case indicates the mutation to disrupt the initiation codon.	
