# Supplementary material for: CELF Family RNA–Binding Protein UNC-75 Regulates Two Sets of Mutually Exclusive Exons of the unc-32 Gene in Neuron-Specific Manners in Caenorhabditis elegans
Source: PLoS Genet. 2013 Feb 28;9(2):e1003337. doi: 10.1371/journal.pgen.1003337 (PMC3585155; doi:10.1371/journal.pgen.1003337)
Supplement: Table S2 — Sequences of the primers used to detect the unc-32 and unc-75 RNAs in RT-PCR assays. (RTF) [file pgen.1003337.s009.rtf]

Table S2. Sequences of the primers used to detect the unc-32 and unc-75 RNAs in RT-PCR assays. 	
Primers used to detect the mature mRNAs and partially spliced RNAs derived from the unc-32 reporter minigenes	
Name	Sequence	Target mRNAs	
attB1 forward	5�f-GGGGACAAGTTTGTACAAAAAAGCAGGCT-3�f	UNC-32E7a-EGFP, UNC-32-E7b-mRFP	
UNC-32#48	5�f-ATGGTGGCTCTAGATTCTGC-3�f	UNC-32E7a-EGFP, UNC-32-E7b-mRFP	
	
Primers used to detect the mature mRNAs and partially spliced RNAs derived from the endogenous unc-32 gene	
Name	Sequence	Position & Direction	
UNC-32#1	5�f-CAGAGCTCACAGAGCTG-3�f	Exon 3, Forward	
UNC-32#37	5�f-TCGAGAACGAACTTCGCGAG-3�f	Exon 3, Forward	
UNC-32#8	5�f-CCGTTGAATTACACCAGCAAC-3�f	Exon 5, Reverse	
UNC-32#38	5�f-CATTACCACGACACGCTCTC-3�f	Exon 5, Reverse	
UNC-32#49	5�f-TCCCTTCTCATCGGTCTCATC-3�f	Exon 6, Forward	
UNC-32#50	5�f-TGCCCATGTCCGTGACCACTT-3�f	Exon 8, Reverse	
	
Primers used to detect the partially spliced unc-32 RNAs	
Name	Sequence	Position & Direction	
unc-32#79	5�f-GTAGTATGTTTTGTGCCTCACC-3�f	Intron 3, Forward	
unc-32#82	5�f-AAAGACACCACCATCACAATACA-3�f	Intron 4c, Reverse	
unc-32#77	5�f-caggtgtttggatgactgatg-3�f	Intron 6, Forward	
unc-32#78	5�f-CTACCCATCACCAATTGTTTACC-3�f	Intron 7b, Reverse	
	
Primers used to detect the mature mRNAs from the endogenous unc-75 gene	
Name	Sequence	Position & Direction	
UNC-75#5	5�f-CACATGCTCAGACTGAGAATCCTG-3�f	Exon 6, Forward	
UNC-75#4	5�f-TACATGCTACAGGTGTTAGGGTAC-3�f	Exon 9, Reverse	
