# Supplementary material for: CELF Family RNA–Binding Protein UNC-75 Regulates Two Sets of Mutually Exclusive Exons of the unc-32 Gene in Neuron-Specific Manners in Caenorhabditis elegans
Source: PLoS Genet. 2013 Feb 28;9(2):e1003337. doi: 10.1371/journal.pgen.1003337 (PMC3585155; doi:10.1371/journal.pgen.1003337)
Supplement: Table S3 — Sequences of the primers used in constructing the UNC-75, ASD-1 and FOX-1 expression vectors. (RTF) [file pgen.1003337.s010.rtf]

Table S3. Sequences of the primers used in constructing the UNC-75, ASD-1 and FOX-1 expression vectors. 	
Primers used to amplify the cDNA fragments	
Sequence	Constructed Entry vectors	
5'-CACCATGGGGCAGCAGCAGCACGAG-3'	pENTR-UNC-75, pENTR-UNC-75(1-114)	
5'-TCAATAAGGACGGCTCTCATTTCT-3'	pENTR-UNC-75, pENTR-UNC-75(417-514)	
5'-TTACGGACGGCTGTCTGTGTCTGC-3'	pENTR-UNC-75(1-114)	
5'-CACCATGGACGACAAGAAGCTTTTC-3'	pENTR-UNC-75(121-246)	
5'-TTATGATTGTTGCTGTAGAACCTG-3'	pENTR-UNC-75(121-246)	
5'-CACCATGGTGACACCCAGAGAAG-3'	pENTR-UNC-75(417-514)	
5'-CACCATGACAACGGCGGCTGAAATAAT-3'	pENTR-ASD-1(1-388)	
5'-TTATTTATTGTGAACTCTCTGAGTAGC-3'	pENTR-ASD-1(1-388)	
5'-CACCATGCAAGCCCTGTACCAACTGTCTGC-3'	pENTR-FOX-1(1-408)	
5'-TTACGTTCTAAGCGCCGGGTT-3'	pENTR-FOX-1(1-408)	
Underlines indicate the sequences for directional TOPO-cloning with pENTR/D-TOPO (Invitrogen). 	
	
Primers used for the mutagenesis	
Sequence	Constructed vectors	
5'-GCTGAAACGTCCGgcAAATGAGAGCCGTgCTgcTTGAAAGGGTGGG-3'	pENTR-UNC-75(AAA)	
5'-CCCACCCTTTCAAgcAGcACGGCTCTCATTTgcCGGACGTTTCAGC-3'	pENTR-UNC-75(AAA)	
5'-AAATCGGCGAATGtAACAGATGGCTGC-3'	pCold-FLAG-UNC-75(121-213)	
5'-GCAGCCATCTGTTaCATTCGCCGATTT-3'	pCold-FLAG-UNC-75(121-213)	
5'-ATTTGAGCAATTTaGTAAAATATATGA-3'	pENTR-UNC-75(G53S)	
5'-TCATATATTTTACtAAATTGCTCAAAT-3'	pENTR-UNC-75(G53S)	
5'-GGAGCATCCAAAGaGTGTGCATTTGTG-3'	pENTR-UNC-75(G165E)	
5'-CACAAATGCACACtCTTTGGATGCTCC-3'	pENTR-UNC-75(G165E)	
5'-CGATGGCTGCAACtTTTTCATCTATCA-3'	pENTR-UNC-75(L431F)	
5'-TGATAGATGAAAAaGTTGCAGCCATCG-3'	pENTR-UNC-75(L431F)	
Lowercase indicates the nucleotides different from the wild type. 	
