# Supplementary material for: CELF Family RNA–Binding Protein UNC-75 Regulates Two Sets of Mutually Exclusive Exons of the unc-32 Gene in Neuron-Specific Manners in Caenorhabditis elegans
Source: PLoS Genet. 2013 Feb 28;9(2):e1003337. doi: 10.1371/journal.pgen.1003337 (PMC3585155; doi:10.1371/journal.pgen.1003337)
Supplement: Table S4 — Sequences of the oligo DNAs used in in vitro transcription. (RTF) [file pgen.1003337.s011.rtf]

Table S4. Sequences of the oligo DNAs used in in vitro transcription. 	
Oligo DNAs used in preparing the radiolabelled RNA probes for the EMSAs	
Name	Sequence	Probes	
T7p(+)	5�f- TAATACGACTCACTATAGGGAGA-3�f	All	
unc-32#112/T7(-)	5�f-ACAATCATGCAAAAACTACCCATCACCTCTCCCTATAGTGAGTCGTATTA-3�f	WT intron 7b	
unc-32#114/T7(-)	5�f-ACAATCATcgAAAAACTACCCATCACCTCTCCCTATAGTGAGTCGTATTA-3�f	Mut intron 7b	
unc-32#102/T7(-)	5�f-ACAACACAACAAAAAGTAACGGGACAGTAGAAGGCGGGTGCGTGCTAATCTCCCTATAGTGAGTCGTATTA-3�f	2-1-1	
unc-32#104/T7(-)	5�f-ACAACACAACAAAAAGTAACGGGACAGTAGAAGGCGGGTtttTttTAATCTCCCTATAGTGAGTCGTATTA-3�f	2-1-1a	
unc-32#106/T7(-)	5�f-ACAACACAACAAAAAGTAACGGGACAGTAGAAttttttTGCGTGCTAATCTCCCTATAGTGAGTCGTATTA-3�f	2-1-1b	
unc-32#122/T7(-)	5�f-ACAACACAACAAAAAGTAACGGGAtAtTAtAAGGCGGGTGCGTGCTAATCTCCCTATAGTGAGTCGTATTA-3�f	2-1-1c	
unc-32#124/T7(-)	5�f-ACAACACAACAAAAAtTAAttttACAGTAGAAGGCGGGTGCGTGCTAATCTCCCTATAGTGAGTCGTATTA-3�f	2-1-1d	
unc-32#108/T7(-)	5�f-AtAAtAtAAtAAAAAGTAACGGGACAGTAGAAGGCGGGTGCGTGCTAATCTCCCTATAGTGAGTCGTATTA-3�f	2-1-1e	
Underlines indicate the T7 promoters. Lowercase indicates the nucleotides different from the wild type. 		
