# Supplementary material for: CELF Family RNA–Binding Protein UNC-75 Regulates Two Sets of Mutually Exclusive Exons of the unc-32 Gene in Neuron-Specific Manners in Caenorhabditis elegans
Source: PLoS Genet. 2013 Feb 28;9(2):e1003337. doi: 10.1371/journal.pgen.1003337 (PMC3585155; doi:10.1371/journal.pgen.1003337)
Supplement: Table S5 — Sequences of the primers used to prepare the templates for in vitro transcription. (RTF) [file pgen.1003337.s012.rtf]

Table S5. Sequences of the primers used to prepare the templates for in vitro transcription. 	
Name	Sequence	Probes	
UNC-32#45/T7(+)	5'-TAATACGACTCACTATAGGGAGAAGCACTTGGTATCCTGGACAG-3'	1	
unc-32#84	5'-GTAGAAGGCGGGTGCGTG-3'	1	
UNC-32#83/T7(+)	5'-TAATACGACTCACTATAGGGAGAGGGGGGCCATAGACAATTG-3'	2, 2-1	
unc-32#96	5'-TAAAAAGTCGATCTTCTCAC-3'	2-1	
unc-32#95/T7(+)	5'-TAATACGACTCACTATAGGGAGAGTTTTTATTTTGTGAGAAGATCGAC-3'	2-2	
unc-32#98	5'-GCGTCTGGGTTAATATAATCTG-3'	2-2	
unc-32#97/T7(+)	5'-TAATACGACTCACTATAGGGAGACAGATTATATTAACCCAGACGC-3'	2-3	
unc-32#100	5'-GAATTTATGTTATAGATGTTTTATGGTAG-3'	2-3	
unc-32#99/T7(+)	5'-TAATACGACTCACTATAGGGAGACTACCATAAAACATCTATAAC-3'	2-4	
unc-32#46	5'-ACGTGATATTGACCCAGTCAC-3'	2, 2-4	
unc-32#47/T7(+)	5'-TAATACGACTCACTATAGGGAGAGTACTCTTTAATGGCGCACAC-3'	3	
unc-32#78	5'-CTACCCATCACCAATTGTTTACC-3'	3	
unc-32#85/T7(+)	5'-TAATACGACTCACTATAGGGAGACGATCTTCATCAACACCGTCT-3'	4	
UNC-32#16	5'-TGATATCAGCACGAACACTCT-3'	4	
Underlines indicate the T7 promoters. 		
